# miRNA Enriched in Human Neuroblast Nuclei Bind the MAZ Transcription Factor and Their Precursors Contain the MAZ Consensus Motif

**DOI:** 10.3389/fnmol.2017.00259

**Published:** 2017-08-21

**Authors:** Belinda J. Goldie, Chantel Fitzsimmons, Judith Weidenhofer, Joshua R. Atkins, Dan O. Wang, Murray J. Cairns

**Affiliations:** ^1^School of Biomedical Sciences and Pharmacy, The University of Newcastle, Callaghan NSW, Australia; ^2^Centre for Brain and Mental Health Research, Hunter Medical Research Institute, The University of Newcastle, Callaghan NSW, Australia; ^3^World Premier International Research Center – Institute for Integrated Cell-Material Sciences, Kyoto University Kyoto, Japan; ^4^The Keihanshin Consortium for Fostering the Next Generation of Global Leaders in Research Kyoto, Japan

**Keywords:** nuclear miRNA, primate-specific, regulation of gene expression, neuron differentiation, Argonaute proteins, splicing

## Abstract

While the cytoplasmic function of microRNA (miRNA) as post-transcriptional regulators of mRNA has been the subject of significant research effort, their activity in the nucleus is less well characterized. Here we use a human neuronal cell model to show that some mature miRNA are preferentially enriched in the nucleus. These molecules were predominantly primate-specific and contained a sequence motif with homology to the consensus MAZ transcription factor binding element. Precursor miRNA containing this motif were shown to have affinity for MAZ protein in nuclear extract. We then used Ago1/2 RIP-Seq to explore nuclear miRNA-associated mRNA targets. Interestingly, the genes for Ago2-associated transcripts were also significantly enriched with MAZ binding sites and neural function, whereas Ago1-transcripts were associated with general metabolic processes and localized with SC35 spliceosomes. These findings suggest the MAZ transcription factor is associated with miRNA in the nucleus and may influence the regulation of neuronal development through Ago2-associated miRNA induced silencing complexes. The MAZ transcription factor may therefore be important for organizing higher order integration of transcriptional and post-transcriptional processes in primate neurons.

## Introduction

Short non-coding microRNA (miRNA) provide a specificity mechanism for the post-transcriptional management of mRNA in complex eukaryotic cells. By mediating interaction between the RNA-Induced Silencing Complex (RISC) and target mRNAs, miRNA can potentially regulate many aspects of their targets’ post-transcriptional life and death, including the modulation of translational activity or mRNA degradation. At the core of the RISC is a member of the Argonaute (Ago) family of proteins, which associates with the miRNA and orients it for target recognition. Human cells have four Ago isoforms (Ago1-4) of which Ago2 is structurally unique, having endonuclease activity conferred by a catalytic slicer domain. Thus, although it may participate in transcript silencing in a similar manner to Ago1, Ago3, and Ago4, Ago2 exclusively may induce transcript degradation via cleavage of its target mRNAs (Meister et al., 2004). Ago2 can also cleave miRNA precursors to produce mature miRNA independently of Dicer (Diederichs and Haber, 2007).

In the canonical biogenesis pathway, miRNAs are transcribed from genomic loci as long primary strands that undergo cleavage to precursor hairpins (pre-miRs) in the nucleus before being exported to the cytoplasm where they are further cleaved into their mature form and loaded into the RISC. As a result, the repressive functions of the RISC, as well as mature miRNAs themselves, have been primarily associated with the cytosol. However, miRNA and Ago proteins have been detected in the nucleus in a variety of cell types (Hwang et al., 2007; Politz et al., 2009; Jeffries et al., 2011), where they undertake a broader functional repertoire. By participating in the RNA-induced transcriptional silencing (RITS) complex, Ago1 supports the modulation of heterochromatin levels to regulate transcription (Noma et al., 2004). In fact, both Ago1 and Ago2 have been shown to interact with several chromatin remodeling factors in an RNA-dependent manner (Castanotto et al., 2009), while Ago2 is known to directly regulate the SWI/SNF transcription complex at transcription start sites (Carissimi et al., 2015), suggesting multiple mechanisms by which the RISC may influence transcription. Nuclear miRNA may also influence non-coding RNA activity by targeting endogenous anti-sense and sense transcripts, such as miR-671-directed cleavage of CDR1 anti-sense transcripts by Ago2 (Hansen et al., 2011); while various co-regulatory transcriptional relationships between intronic miRNA and their host genes have been described (Ballarino et al., 2009; Janas et al., 2011). Contributions to alternative splicing have been revealed for Ago2 in Drosophila (Taliaferro et al., 2013), and Ago1 in mammalian cells (Allo et al., 2014). Despite this emerging knowledge, the underlying mechanisms governing nuclear miRNA localization and the role of the RISC in nuclear processes such as splicing are generally poorly understood (Salmanidis et al., 2014).

Here we report that a group of mature miRNA are enriched in the nucleus of undifferentiated SH-SY5Y human neuroblastoma, and identify their target genes associated with the RISC proteins Ago1 and Ago2. Most of the nucleus-enriched miRNA were primate-specific and shared a common sequence motif with similarity to the recognition element of the MAZ transcription factor. We also demonstrate that MAZ protein present in nuclear extract can bind pre-miRNA of these nucleus-enriched molecules. Strikingly, the Ago2 associated target genes were also highly enriched with MAZ regulatory elements and functional annotation related to neurons. In contrast Ago1 targets were associated with splicing factor SC35 suggesting these nucleus-enriched miRNAs are more involved with the regulation of splicing.

## Results

### Mature miRNA Preferentially Localized to the Nucleus Are Largely Primate-Specific

**FIGURE 1:**
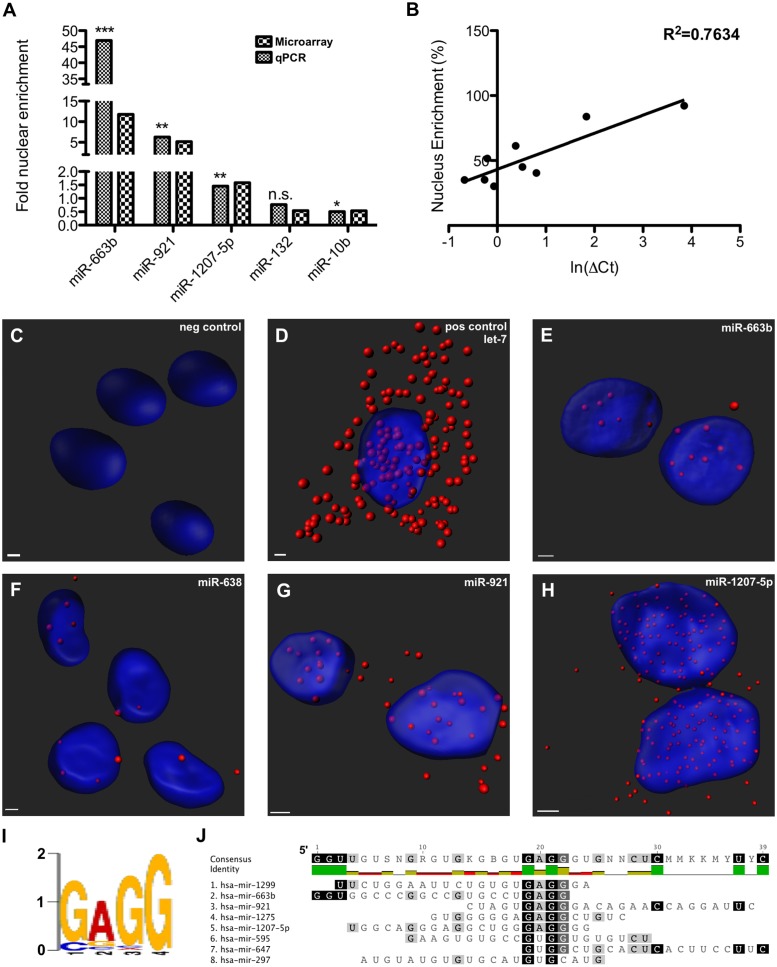
Nucleus-enriched mature microRNA (miRNA) share a common motif. **(A)** Nucleus enrichment of human miRNAs was measured by microarray and confirmed by qPCR. Asterisks indicate significance of the difference between nuclear and cytoplasmic expression (^∗^*p* < 0.05; ^∗∗^*p* < 0.01; ^∗∗∗^*p* < 0.0001). Note the split y-axes. **(B)** A strong correlation observed between raw % nuclear enrichment and Ct value served to validate the analysis. **(C–H)** miRNA FISH detection of nuclear miRNA enrichment. Z-stack confocal images, taken at 63X magnification with oil-immersion on a Zeiss LSM800, were rendered with Imaris software. **(I)** *De novo* motif detected among nucleus-enriched mature miRNA. **(J)** Alignment showing position of motif in miRNA sequence.

Nuclear and cytoplasmic fractions were prepared in triplicate from SH-SY5Y cells by hypo-osmotic lysis, and expression of 847 human miRNA was measured by microarray. Differential enrichment was determined by calculating the nuclear percentage of total raw expression (**Figures 1A,B**). We found that many miRNA have at least some expression in the nucleus (Supplementary Table S1), however, there were 13 miRNAs comprising more than 70% of total mature expression in the nucleus; interestingly most of these were not conserved (**Table 1**). Nuclear enrichment of a subset of these miRNAs was confirmed visually by fluorescent *in situ* hybridization (miRNA-FISH, **Figures 1C–H**). Compared with the let-7a positive control probe (**Figure 1D**), which demonstrated predominantly cytoplasmic signal, the miRNA tested showed unequivocal nuclear enrichment (**Figures 1E–H**). Moreover, the percentages of nuclear signals closely matched those calculated from the microarray data for all probes assayed (**Table 2**). It is possible that miRNA FISH probes display some cross hybridization with very closely related family members.

**Table 1:** MicroRNA (miRNA) with highest % nuclear expression.

miRNA name	Nuclear %	Conservation^∗^
hsa-miR-768-5pˆ	94.17	n/a
hsa-miR-768-3pˆ	93.71	n/a
hsa-miR-1299	93.17	No (hsa, ptr, ppy)
hsa-miR-297	92.72	Yes
hsa-miR-663b	92.17	No (hsa, ptr, ppy, oga, bta)
hsa-miR-647	89.19	No (hsa, ppy)
hsa-miR-595	88.09	No (hsa, ptr, mml, ppy)
hsa-miR-921	83.82	No (hsa, ppy, efu)
hsa-miR-593^∗^	80.62	No (hsa, ptr, mml, ppy)
hsa-miR-1183	78.28	No (hsa, ptr, ppy)
hsa-miR-664^∗^	75.44	Yes
hsa-miR-1275	73.87	No (hsa, ptr, ppy)
hsa-miR-574-5p	71.94	Yes


*^∗^**Species identifiers**: hsa, Homo sapiens; ptr, Pan troglodytes; mml, Macaca mulatta; ppy, Pongo pygmaeus; efu, Eptesicus fuscus; Bta, Bos Taurus; oga, Otolemur garnettii. Conservation determined using miRViewer database (Kiezun et al., 2012)*.

**Table 2:** Nuclear enrichment by microarray and miRNA-FISH.

miRNA name	Microarray %	miRNA-FISH %
hsa-let-7a	46	25
hsa-miR-638	57	62
hsa-miR-663b	92	87
hsa-miR-921	83	66
hsa-miR-1207-5p	61	79


### Nuclear Primate-Specific miRNA Contain a Motif Similar to the Recognition Element of Transcription Factor MAZ

Internal sequence “signals” are often responsible for determining localization of mRNAs, and it has previously been reported that miR-29b is imported into the nucleus under direction of a hexanucleotide element (Hwang et al., 2007). We therefore wondered whether the same or similar sequence might exist among nucleus-enriched miRNA identified by this study. While the aforementioned hexanucleotide was not observed, a recurrent four-nucleotide motif was found within the most nucleus-enriched miRNA, with all but one miRNA containing “GAGG” (**Figures 1I,J**). The only discordant miRNA, miR-297, contained the slightly divergent “GUGC,” and stands out as the only conserved miRNA among those analyzed for the motif. While the motif is relatively common in human miRNA (25%), none of the 30 miRNA least abundant in the nucleus contained this motif (Supplementary Table S2). Statistical support for the distribution bias was provided through contingency analysis, with the motif being found to be significantly associated with nuclear enrichment (*p* < 0.0001, Fisher’s exact test).

It is interesting to note that the “GAGG” motif detected here is similar to the GA-box motif recognized in DNA by the transcription factor Myc-associated zinc finger protein (MAZ), which has been shown to bind to sequences containing “G2AG2” to “G6AG6” (Bossone et al., 1992). Investigation of the pre-miR hairpin sequences for the motif-containing miRNA found putative MAZ binding elements “GGGAGGG” in pre-miR-1207 and pre-miR-647, and the shorter “GGAGG” in pre-miR-595. We therefore proceeded to investigate the possibility of interactions between MAZ and pre-miRNA in the nucleus.

### MAZ Binds to Precursor miRNA in the Nucleus

**FIGURE 2:**
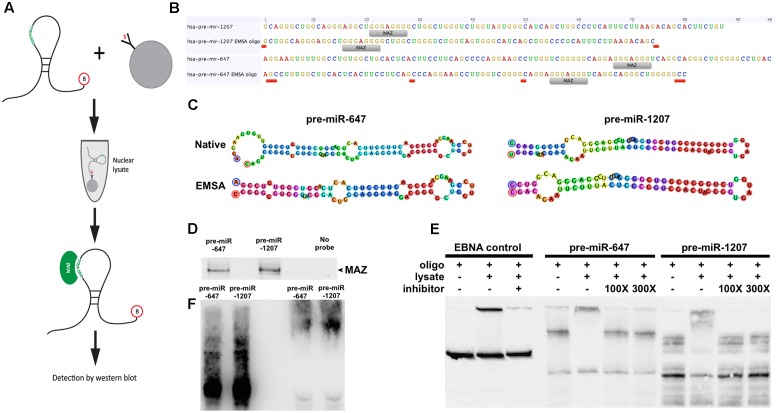
MAZ binds to pre-miR-1207 and -647 in the nucleus. **(A)** Schematic illustration of the workflow of the RNA pull-down assay. Biotinylated pre-miRNA were conjugated to streptavidin-coated beads and used as bait to retrieve binding proteins from pre-cleared nuclear lysate. **(B)** Design of pre-miR oligos for electrophoretic mobility shift assay (EMSA). “MAZ” labels indicate putative MAZ binding sites. Red labels indicate where native sequences were edited to achieve the 80 nt length limit. **(C)** Predicted hairpin structures of native and edited EMSA pre-miRs. Bulge loop features from the original predictions were retained in the new sequences. Oligo design and structure prediction was done using Geneious software. **(D)** Western blot detection of RNA pulldown assay. MAZ was detected at 55 kDa. **(E)** EMSA demonstrating shift of pre-miRs by addition of nuclear lysate. Me1a1 oligo was used as a competitive inhibitor to binding, which reversed the observed shift. **(F)** Western-EMSA (WEMSA) shows the presence of MAZ in the shifted band.

To test whether MAZ can bind precursor miRNA in the nucleus we performed an RNA pull-down assay using 3′-biotinylated pre-miRs as bait (**Figure 2A**). Due to length constraints in the oligonucleotide synthesis process, modified pre-miR-1207 and -647 were designed to have no more than 80nt while retaining predicted secondary structure features (**Figures 2B,C**). Pre-miR:protein complexes were purified from pre-cleared nuclear lysates using streptavidin-coated beads and resolved by SDS–PAGE. The 55 kDa MAZ protein was detected in relation to both precursors by western blot (**Figure 2D**). To further support interaction with the MAZ binding sequence, we used an electrophoretic mobility shift assay (EMSA). We observed that the addition of nuclear lysate caused a shift in the migration of both pre-miRs that was reversible in the presence of a known MAZ binding control sequence Me1a1 (Komatsu et al., 1997) as a competitive inhibitor at both 100- and 300-fold excess (**Figure 2E**). The specificity of the MAZ:pre-miR interaction was also confirmed by immuno-labeling the super-shifted complex with the MAZ antibody after transfer to nitrocellulose (WMSA) (**Figure 2F**).

### Nuclear Ago1 and Ago2 Have Differential Distribution and Functional Targets

**FIGURE 3:**
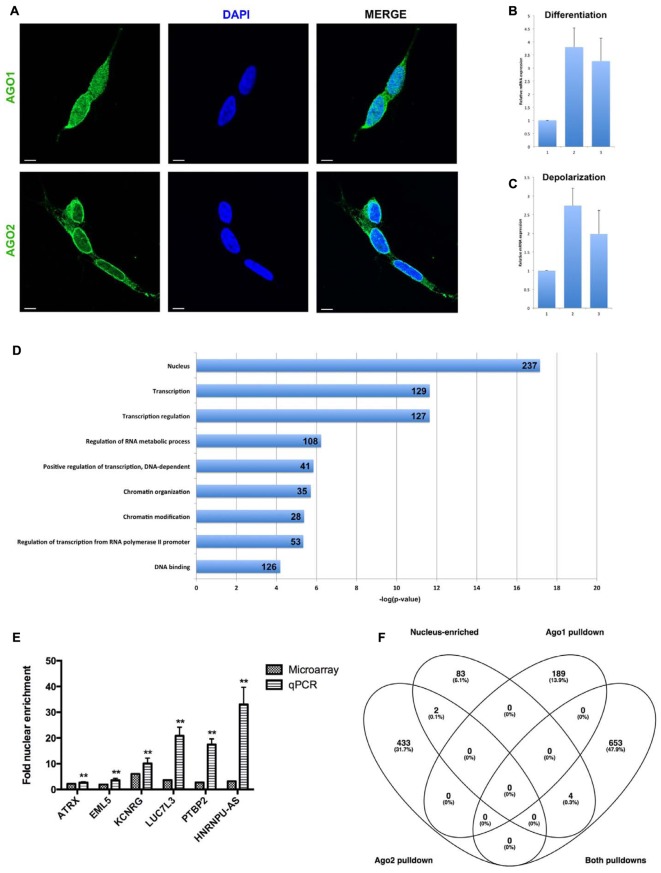
Analysis of nuclear mRNA by Argonaute (Ago)1/2 RIP-seq and exon microarray. **(A)** Ago1 and Ago2 are differentially distributed in the nucleus. Single slice images were taken on a Zeiss LSM800 confocal microscope at 63X magnification with oil-immersion. Scale bar, 5 μm. **(B)** qPCR detection of MAZ mRNA abundance in response to sequential ATRA and BDNF differentiation of SH-SY5Y cells. **(C)** qPCR detection of MAZ mRNA abundance in response potassium-induced depolarization of differentiated SH-SY5Y cells. **(D)** Functional analysis of mRNAs pulled down by both Ago1 and Ago2 showed strong relation to transcriptional regulation. **(E)** Taqman qPCR validation of microarray-detected nuclear mRNA enrichment. **(F)** Nucleus-enriched mRNAs are not associated with Ago1 or Ago2. ^∗∗^*p* < 0.01.

To determine whether nuclear mature miRNA enrichment may be associated with canonical Ago-mediated mechanisms, we first performed immunocytochemistry to visualize the distribution of the RISC core components Ago1 and Ago2. Both Ago proteins were detected in the nucleus, however, Ago1 demonstrated a granular distribution throughout the nucleoplasm, while Ago2 was more heterogeneous and appeared particularly localized adjacent to the nuclear envelope (**Figure 3A**). In addition to these distinct localization patterns, these related proteins have inherent structural differences: Ago2 contains a slicer domain and is associated with degradation, while Ago1 lacks this domain and is associated with translational repression (Meister, 2013). We therefore hypothesized their roles in nuclear miRNA function would likewise be divergent.

To investigate the miRISC function of nucleus-enriched miRNA we performed RNA immunoprecipitation on nuclear lysates followed by RNA-Seq of Ago1- and Ago2-associated transcripts. A comprehensive list of miRNAs targeting positive reads was filtered for nucleus-enriched miRNAs and compared between samples, yielding 189 mRNAs unique to Ago1, 435 unique to Ago2 and 657 transcripts detected in both pull-downs (Supplementary Table S3); these lists were functionally analyzed using Gene Annotation Tool to Help Explain Relationships (GATHER) and the DAVID Functional Annotation Clustering (FAC) tool.

In these undifferentiated neuronal cells Ago2-associated, nuclear miRNA targeted transcripts were significantly enriched with neuronal terms including axonogenesis (*p* < 0.0001) and neurogenesis (*p* = 0.0003), while the most significant FAC clusters (ES = 3.9 and ES = 3.2) related to neuron development, neuron differentiation, neuron projection, axonogenesis, and axon guidance. Since Ago2 is linked to target degradation, this suggests the possibility of miRNA-mediated nuclear sequestration of transcripts not yet required in these immature cells. Genes contributing to this functionality included the plasticity-associated cytoskeleton regulator ARC, glutamate receptors GRIA3 and GRIA4, and axon guidance receptors ROBO1 and ROBO2. In contrast, mRNAs associated with Ago1 were functionally linked to more general cellular processes and enrichment of functional domains, such as cell adhesion (*p* = 0.05), actin cytoskeleton organization (*p* = 0.004), and regulation of kinase activity (*p* = 0.015). Association of these mRNAs with translational repressor Ago1 is consistent with the ongoing, but time-sensitive, requirement of their coded proteins.

Intriguingly, a transcription factor analysis also carried out using GATHER identified MAZ as a common driver (Bayes factor = 20) of 301 of the 435 genes uniquely associated with Ago2, however, it was not linked to transcripts unique to Ago1 nor those common to both. We decided to investigate this relationship between MAZ and neuronal function by measuring MAZ expression changes in response to cues for differentiation and depolarization. Sequential treatment with ATRA and BDNF induced a significant, threefold increase in MAZ mRNA abundance (**Figure 3B**), while a single depolarization event evoked a further threefold induction (**Figure 3C**). Taken together these results support a co-operative relationship between MAZ and Ago2 in supporting neuron development and mature neuronal function.

### Convergent Functions of Ago1/2 in Transcriptional Regulation

Transcripts common to both Ago1 and Ago2 were very strongly linked by FAC to the nucleus (ES = 6.3), transcriptional regulation (ES = 6.4), and chromatin modification (ES = 4.5) (**Figure 3D**). This is consistent with a recent report demonstrating a role for Ago1 in binding transcriptional enhancers and as an important regulator of splicing efficiency (Allo et al., 2014). Also enriched in this list were molecules containing RNA recognition motifs capable of binding RNAs (ES = 5.2). GATHER identified gene ontologies including GPCR signaling, protein modification, and nucleotide metabolism (all *p* < 0.0001). Important among this list was LIN28A, the human homolog of pluripotency factor Lin-28. This transcription factor contains both DNA and RNA binding domains, and has been shown to interfere with pre-miR processing, both negatively regulating pre-let-7 maturation and being itself negatively regulated by the mature let-7 to control differentiation of neural stem cells (Rybak et al., 2008).

### Nucleus-Enriched mRNAs Are Independent of Ago

In addition to analysis of Ago-associated nuclear transcripts, we sought to obtain a picture of overall nuclear expression and enrichment. Total RNA was analyzed on Affymetrix Human Exon 1.0 ST microarrays and the resultant data summarized for both gene-level and exon-level expression. Nucleus-enriched mRNAs were identified in the same manner as for miRNA, yielding a group of 91 transcripts having greater proportional abundance in the nucleus (compared with 2035 with cytoplasmic enrichment). A panel of these transcripts was verified as nucleus-enriched by Taqman qPCR analysis (**Figure 3E**). Strikingly, there was negligible overlap between these nucleus-enriched mRNAs and those associated with Ago1, Ago2 or both (**Figure 3F**), suggesting a different functional purpose for their sequestration. The small list size limited the significance of the ontological analysis here; the largest proportions of the gene list were associated with RNA binding (11/91, *p* = 0.02) and the cytoskeleton (10/91, *p* = 0.03).

### Nucleus-Enriched miRNA May Influence Splicing of Genes Involved in Neuron Development and Function via Interaction with Splicing Factor SC35

**FIGURE 4:**
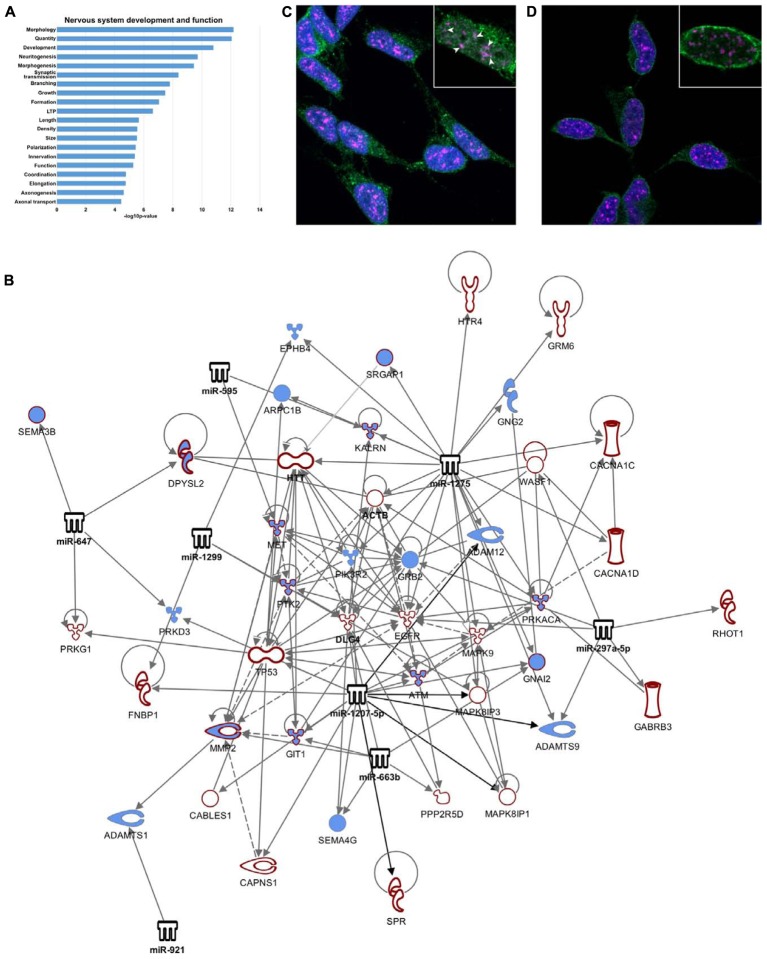
Nucleus-enriched miRNA may regulate splicing via SC35. **(A)** Functional enrichment of transcripts differentially spliced between nucleus and cytoplasm. **(B)** Network diagram depicting nucleus-enriched miRNA targeting of differentially spliced mRNA. Interactions have been filtered to show strong regulation of axon guidance signaling. Diagram was generated using Ingenuity Pathway Analysis (IPA) software. **(C,D)** Single slice images demonstrating nuclear localization of Ago1, Ago2, and SC35, taken on a Zeiss LSM800 confocal microscope at 100X magnification with oil-immersion. **(C)** Ago1 co-localizes with SC35 (SRSF2). Co-localized molecules appear white, demonstrated by arrowheads. All SC35 foci contain at least one Ago1 puncta. **(D)** Ago2 is not co-localized with SC35.

A splicing ANOVA was carried out on exon-level expression data using Genespring GX12 software to identify mRNAs demonstrating significantly different (FDR < 0.01) splicing between nuclear and cytoplasmic compartments. This analysis found 1207 transcripts having splicing index > 2, of which approximately one third (473) were identified by Ingenuity Pathway Analysis (IPA) as targets of the nucleus-enriched miRNA, yielding 637 miRNA-mRNA regulatory pairings. IPA core functional analysis of these genes showed significant enrichment of “nervous system development and function” (*p* = 6.66E-13 – 3.57E-5, **Figure 4A**). Filtering the pairings for “nervous system signaling” identified a tight network comprising 7 miRNA and 54 mRNA, including PSD-95 and beta-actin, that was highly connected with axon guidance signaling (**Figure 4B**). Of these 473 mRNAs, almost 25% were identified as also having nuclear miRNA-mediated Ago-association, prompting us to investigate co-localization of Ago with splicing machinery. Serine and arginine rich splicing factor 2 (SRSF2 or SC35) plays a role in RA-mediated alternative splicing (Apostolatos et al., 2010), and since RA induces neuronal differentiation of SH-SY5Y cells we reasoned that it might contribute to this network. Moreover, DROSHA-DGCR8-associated pri-miRNAs have been reported to accumulate in SC35 positive foci (Pawlicki and Steitz, 2009). We therefore performed immuno-staining to check for co-localization of both Ago1 and Ago2 with SC35. We detected substantial co-localization of Ago1 and SC35 (**Figure 4C**) that was not evident for Ago2 (**Figure 4D**).

## Discussion

In this study we combined genome-wide analysis of nucleus-enriched miRNA with Ago1 and Ago2 RIP-seq and splicing array technologies to obtain a multi-dimensional picture of the post-transcriptional regulatory composition of the nuclear compartment. We showed that MAZ TF can bind to precursor miRNA by identifying a shared motif among nucleus-enriched, primate-specific mature miRs. Piriyapongsa et al. (2011) bioinformatically observed significant enrichment of transcription factor binding sites within the sequences of precursor miRNAs compared with randomized sequence or other genomic regions such as introns, suggesting that direct interaction between pre-miRs and TFs may be an important mechanism of transcriptional regulation (Piriyapongsa et al., 2011). Additionally, our analysis found three non-conserved MREs for mature miRNA derived from the tested pre-miRs predicted in the MAZ 3′ UTR, suggesting the possibility of a more complex auto-regulatory relationship.

**FIGURE 5:**
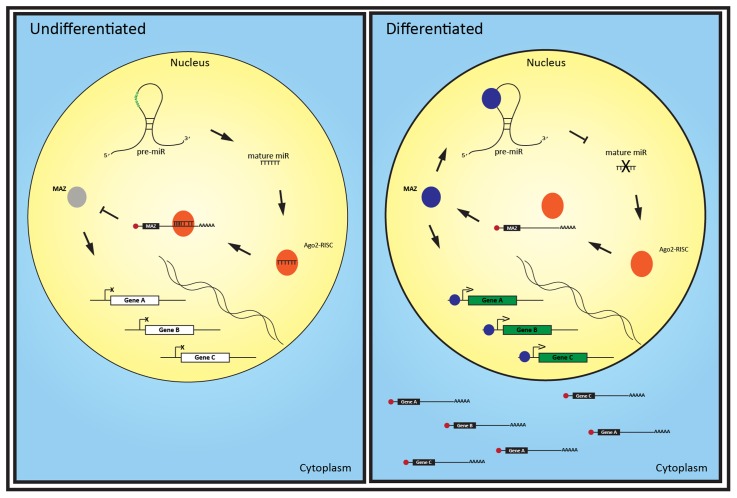
A putative MAZ-miRISC loop regulates neuronal differentiation via nucleus-enriched miRNA. The nuclear expression of a set of recently evolved miRNAs in undifferentiated cells enables Ago2-RISC complexes to down-regulate the production of key neuronal genes and the transcription factor MAZ. As MAZ increases during differentiation, it binds to pre-miRs, potentially inhibiting their maturation. Reduction of these miRNA alleviates repression of MAZ mRNA, further increasing MAZ translation. MAZ protein can then bind to target genes and drive their transcription and, as these transcripts are no longer silenced by the now reduced miRNA, the mature mRNA can be exported to the cytoplasm.

These findings suggest MAZ could be helping to drive neuronal function by modulating Ago2-associated targets of miRNA localized in the nucleus (**Figure 5**). We suspect this miRNA-Ago2-MAZ regulatory circuit is involved in neuronal differentiation in a similar manner to let-7, miR-125, and lin-28 regulation of neural stem cell differentiation (Rybak et al., 2008). In this putative mechanism, pre-miRNA act as a decoy for MAZ protein, down regulating transcription of its targets, while the mature miRNA acts on the 3′ UTR of these nascent mRNA to prevent nuclear export and translation. Interestingly, Ugai et al. (2001) found MAZ throughout the cytoplasm of retinoic acid differentiated PC19 cells, and particularly enriched in the neurites, compared with immature cells where MAZ localized to the nucleus. Thus it is also plausible that MAZ could interact with miRNA or their precursor forms in the periphery of neurons and become trans-located to the nucleus with or without the associated miRNA to drive gene expression response to environmental or developmental cues.

Alternatively, MAZ may be altering or inhibiting Ago2-associated, Dicer-independent miRNA maturation (Diederichs and Haber, 2007). Using the pre-miR-451 system, which is known to be dependent on the slicer activity of Ago2 for maturation, Yang et al. (2012) demonstrated several functional parameters required for Ago2-associated pre-miR processing in human cervical cancer (HeLa) and mouse embryonic fibroblast (MEF) lines. Among our nucleus-enriched miRNA only pre-miR-297 fulfills the requirements of base-pairing at most positions within the hairpin and short hairpin length, and has a uracil at the 5′ position which is associated with more efficient Ago2 pre-miR processing. This is an interesting observation considering that miR-297 was the only conserved miRNA in this list, and miR-451 is itself well conserved, however, neither of these homologs was identified among a list of Ago2-dependent miRNA in the mouse striatum (Schaefer et al., 2010), suggesting that the mechanism, or at least the miRNA, may be cell-type, species or primate-specific.

Mature miRNA in the cytoplasm are known to be associated with Ago1 or Ago2 in the RISC, so we wondered if the mature miRNA enriched in the nucleus are also associated with RISC proteins. A recent study in SH-SY5Y found that overexpression of Ago1, but not Ago2, resulted in slowing of the cell cycle and induction of apoptosis, while over expression of either resulted in significant enhancement of the neuronal phenotype during retinoic acid differentiation (Parisi et al., 2011). This suggests that although there may be some mechanistic convergence between the two Ago proteins, it is likely there will be some functional outcomes unique to each. Our findings support this, demonstrating distinct functional enrichment between Ago1- and Ago2-associated transcripts. Moreover, we showed that the two proteins exhibit very different sub-nuclear localization patterns. Consistent with previous reports, Ago2 was found adjacent to the inner nuclear envelope while Ago1 was distributed throughout the nucleus (Huang et al., 2013); although we now show that Ago1 co-localizes with SC35 foci, suggesting an association with the spliceosome.

MicroRNAs have been linked to splicing regulation through feedback networks involving SF2/ASF in cells including SH-SY5Y (Meseguer et al., 2010; Wu et al., 2010). Since nucleo-cytoplasmic differences between splice variants have been shown to have significant association with differential 3′ UTR exon expression (Chen, 2010), it is plausible that this regulatory relationship also extends to compartmental discrimination. In particular our analysis showed significant neuronal functionality among differentially spliced targets of nucleus-enriched miRNA, and miRNA have been shown to differentially regulate splice variants of key neuronal genes in neuronal cells (Laneve et al., 2007; Guidi et al., 2010).

A similar profiling study in rat neurons detected miRs specifically enriched in the nucleus, but no consensus sequence was found (Khudayberdiev et al., 2013). While these miRNA were not among nucleus-enriched transcripts in our study, we primarily observed non-conserved miRNA associated with primates. Indeed this is an important finding in this human neuronal cell model. A computational study identified 269 primate-specific miRNAs and found that most of these were expressed at low levels in mature tissues (Lin et al., 2010), suggesting an important role for non-conserved miRNAs in temporo-spatial transcript regulation. Moreover, it has been observed that miRNA loci have undergone a greater degree of human-specific developmental remodeling in the brain than other transcript types (Somel et al., 2011). These observations are highlighted by recent studies linking primate-specific miRNAs to major depression (Lopez et al., 2014), Alzheimer’s disease (Zhang et al., 2016), and glioblastoma (Shi et al., 2014).

The functional repertoire of miRNA is rapidly expanding from their initial role as post-transcriptional regulators, and we have demonstrated here and elsewhere that, at least in neurons, subcellular location is just as important for miRNA as for mRNA. Moreover, our findings in the nucleus and neurites have revealed substantial enrichment of primate-specific miRNA in these critical compartments, suggesting these molecules may make a pivotal contribution to the development of higher brain functions. In our view, shifting the focus of miRNA studies from individual gene-miRNA regulatory relationships to systems level analyses, along with broadening consideration from conserved-only targets to include non-conserved, will assist further elucidation of miRNA biology and could facilitate understanding of much that is currently unknown about human cellular complexity.

## Experimental Procedures

### Cell Culture

SH-SY5Y cells were obtained from ATCC. Populations were maintained at 37°C, 5% CO_2_, 90% humidity in DMEM (Hyclone) supplemented with 10% Fetal Bovine Serum (Sigma–Aldrich), 2% HEPES, and 1% L-glutamine (both Hyclone). Cells were routinely passaged and harvested by washing with Phosphate-Buffered Saline (PBS, Gibco) followed by brief incubation with trypsin. Assays were carried out on cells at passage 10.

### Subcellular Fractionation

Cells were harvested and fractionated by hypotonic lysis as described by Wang et al. (2006) with some modifications. Briefly, cells were washed three times with ice-cold PBS and pelleted at 4,000 rpm for 3 min. The pellet was resuspended in 1 ml ice-cold RSB Resuspension Buffer (10 mM Tris, pH 7.4, 10 mM NaCl, 3 mM MgCl_2_), incubated on ice 3 min, and centrifuged as before. The supernatant was removed, pellet volume estimated, then resuspended in 4x volume of RSBG40 Lysis Buffer (10 mM Tris, pH 7.4, 10 mM NaCl, 3 mM MgCl2, 10% glycerol, 0.5% NP-40, 0.5 mM DTT, 100 U/ml RNase inhibitor) by slow pipetting. Nuclei were pelleted at 7,000 rpm for 3 min, and the supernatant kept as the cytoplasmic fraction. Nuclei were resuspended in RSBG40, and 1–10th volume of detergent (3.3% wt/wt sodium deoxycholate, 6.6% vol/vol Tween 40, diluted 1:5) was added by gentle vortexing. Nuclei were pelleted as before, and the supernatant pooled with the cytoplasmic fraction. The nuclear pellet was washed with RSBG40 and collected at 10,000 rpm for 5 min and the pellet used for nuclear RNA extraction. Nuclear integrity was checked by light microscopic examination of samples, diluted 1:2 with trypan blue, at 40X magnification. Whole cells from the same samples were used as controls where appropriate.

### RNA Extraction, Quantification, and Quality Assessment

Total RNA was extracted using TRIzol reagent per manufacturer’s instructions (Invitrogen), with the modification of 2 μl of glycogen (20 mg/ml, Sigma) added to the isopropanol precipitation step, which was allowed to proceed overnight at -30°C. The following day, samples were centrifuged for 30 min at 10,000 rpm, 4°C, before completion of the standard procedure. Purified RNA was quantified using the Qubit fluorometer and Quant-IT RNA assay kit per manufacturer’s instructions (Invitrogen). RNA quality was checked using Bioanalyzer RNA 6000 Nano chips per manufacturer’s instructions (Agilent).

### Genome-Wide Analysis of miRNA Expression

Total RNA was labeled using a FlashTag Biotin HSR RNA labeling kit according to manufacturer’s instructions (Genisphere). Labeled RNA was hybridized to Genechip miRNA 1.0 microarrays, washed, stained, and scanned per the manufacturer’s instructions (Affymetrix).

### Genome-Wide Analysis of Gene Expression

Total RNA was transcribed to cDNA and amplified using the Applause WT-Amp Plus ST kit, then fragmented and labeled with the Encore Biotin module, both according to the manufacturer’s instructions (NuGEN). Labeled cDNA was hybridized to GeneChip Exon 1.0 ST microarrays, washed, stained, and scanned as above. Data analyses were conducted at gene (as described below) and exon expression (splicing, using Genespring 12) levels.

### Analysis of Microarray Expression from Different Subcellular Compartments

We expected great variation between nuclear and cytoplasmic expression, and it has not been well established whether normalization would preserve or mask the differences. We therefore undertook to analyze the data by three methods and perform validation on candidate miRNA from each.

Method 1 – RMA (across-sample) normalization. Data were normalized using the Affymetrix standard Robust Multichip Algorithm (RMA) with *p*-value correction for multiple testing, and differentially compartmentalized genes were identified by empirical linear Bayesian model (Smyth, 2004).

Method 2 – Signal rank (in-sample) normalization. Data were analyzed as recently described by Jeffries et al. (2011) for nucleus-cytoplasmic fractionation of NPCs. Briefly, probe signals from each sample were sorted in descending order and a rank calculated based on the distance of each signal from the median signal in that sample. Compartmental enrichment was defined as every sample from one compartment having higher rank than every sample from the other.

Method 3 – No normalization. Raw signals from each sample were averaged across replicates, and probes with low expression in all samples (raw signal < 30) removed. The nuclear proportion of expression was calculated as the percentage that nuclear signal contributed to total array signal from each condition (nucleus + cytoplasm). A miRNA was considered enriched in the nucleus if its “Nuclear % expression” was greater than 70%.

### Quantitative Real-Time PCR (qPCR)

For validation of miRNA expression, multiplex reverse transcription was performed on 500 ng of DNaseI-treated total RNA using oligo (dT) primer and, where appropriate, specific primers for individual miRNAs. All primers were added to a final concentration of 40 nM. Reactions were performed using Superscript II reverse transcriptase in 5X first-strand buffer per manufacturer’s instructions (Invitrogen). Real-time PCR was performed in triplicate on diluted cDNA (1:40) combined with Power SybrGreen master mix (Applied Biosystems) with 10 μM of the appropriate forward and reverse primers, in a final volume of 12.5 μl, using an ABI prism 7500 sequence detection system (Applied Biosystems). Forty cycles of PCR were applied; for gene expression, the annealing temperature was set at 60°C, while for miRNA the annealing step was at 50°C.

Gene expression was confirmed using Taqman pre-designed qPCR assays per the manufacturer’s protocol (Life Technologies).

### Functional Integration of miRNA and Gene Expression

A list of nucleus-enriched miRNA was loaded into IPA software. A target filter analysis was performed on miRNA to identify target mRNA; this list was refined by pairing with nucleus-enriched mRNA, or mRNAs demonstrating significantly different splicing between nucleus and cytoplasm, and Core Analysis performed on gene lists generated from target gene-miRNA pairings.

### miRNA Fluorescent *In Situ* Hybridization (miRNA-FISH)

Cells were seeded in 96-well optical glass-bottom plates (Nunc). After 24 h, the QuantiGene viewRNA miRNA ISH Cell Assay was performed according to the manufacturer’s protocol (Affymetrix), with minor modifications. Collagen was substituted for poly-L lysine as the coating substrate to promote adhesion and preserve morphology of this cell line; coating was for 2 h at 37°C followed by 1 h incubation at 37°C with growth medium before seeding. Additionally, PFA fixation was performed without prior PBS washing to ensure maintained adhesion. Z-stack images were obtained using a Zeiss LSM-800 confocal microscope with 63X oil-immersion objective, and 3D rendering and spot analysis performed in Imaris software.

### RNA-Binding Assay

160 pmol of 3′-biotinylated pre-mir-647 and pre-mir-1207 were bound to 2 mg of streptavidin-coated magnetic beads (M-280, Invitrogen) in binding buffer (10 mM Tris HCl pH 7.5, 1 mM EDTA, 2 M NaCl), according to the manufacturers instructions. Nuclear lysate (400 mg) was pre-cleared with 1 mg of streptavidin-coated magnetic beads prior to incubation with the probe-bead complex in the presence of 200 μl of BS/THES buffer [22 mM Tris HCl pH 7.5, 10 mM HEPES, 4.4 mM EDTA, 8.9% Sucrose (mass/vol), 62 mM NaCl, 5 mM CaCl_2_, 50 mM KCL, 12% glycerol, 0.3% protease inhibitor; sterilized with 0.2 μM filter]. After a 30 min incubation at room temperature and removal of unbound supernatant, the beads were washed three times with BS/THES buffer containing 10 μg/ml Poly dI-dC and then twice with BS/THES buffer alone to remove non-specifically bound proteins. Elution of specifically bound proteins was performed by re-suspending the beads in 40 μl of SDS sample buffer and boiling for 5 min. The recovered fraction was analyzed by SDS–PAGE, on 4–15% polyacrylamide gels, as described above. Control binding assays using beads without the probe were also included.

### Electrophoretic Mobility Shift Assay (EMSA)

Nuclei were isolated as above, the pellet re-suspended in RIPA buffer (50 mM Tris-HCL pH 8.0, 150 mM NaCl, 0.5% sodium deoxycholate, 0.1% SDS, 1% NP-40, 1000 μM 4-(2-aminoethyl) benzenesulfonyl fluoride (AEBSF), 0.8 μM pepstatin A, 20 μM E-64, 40 μM bestatin, 15 μM leupeptin, and 15 μM aprotinin) and incubated on ice for 30 min. The supernatant was retained as the nuclei fraction after centrifugation (13,000 rpm, 15 min, 4°C). Protein concentration was determined using a bicinchoninic acid assay (BCA).

The 20 μl Epstein-Barr nuclear antigen (EBNA) control binding reaction included 50 ng/μg of Poly (dI^∗^dC), 1x binding buffer (100 mM Tris, 500 mM KCl, 10 mM DTT), 2.5% glycerol, 5 mM MgCl_2_, 0.05% NP-40, 1 unit EBNA extract and 20 fmol of Biotin-EBNA control DNA and 4 pmol (200-fold molar excess) unlabeled EBNA DNA. The 20 μl EMSA binding reaction included 50 ng/μg of Poly (dI^∗^dC), 1x binding buffer, 2.5% glycerol, 5 μg nuclear lysate and 20 fmol biotin-mir-647 or biotin-mir-1207. For competition assays double-stranded Me1a1 (1–4 pmol; 50- to 200-fold excess) was used as an unlabeled competitor. For super shift assays 5 μg anti-MAZ antibody was added to the reaction mixture. The reaction was incubated at RT for 20 min. For competitor assays, Me1A1 was added prior to lysate and incubated for 20 min at RT. For super shift assays, the antibody was added after the lysate and incubated for an additional 20 min at RT. The reactions were resolved on a non-denaturing 6% polyacrylamide gel, that had been pre-run at 300 V for 1 h in 0.5% TBE buffer, at 380 mA for 2 h. Samples were semi-dry transferred using 0.5x TBE to a nylon membrane at 380 mA for 30 min. The transferred complex was cross-linked to the membrane using 254 nm bulbs on the 60 s auto exposure crosslink function. The biotin-labeled RNA was detected by chemiluminescence, according to the manufactures instructions (Thermo Scientific) and imaged on the FUJI LAS 1000 imaging system (FUJIFILM Life Science, Stamford, CT, United States).

### Immunofluorescence

Cells, plated on collagen-coated cover glass, were fixed with 4% PFA then permeabilized with 0.01% TritonX-100. After blocking with 5% goat serum, incubation with primary antibodies rabbit anti-Ago1 (ProteinTech 19690-1-AP), rabbit anti-Ago2 (Abcam ab156970), and mouse anti-SC35 (Abcam ab11826) was overnight at 4°C. Appropriate secondary antibodies were selected from the Alexa-color series (Invitrogen). Finally, samples were stained with DAPI then mounted using ProLong Diamond (Invitrogen). Z-stack confocal images were obtained using a Zeiss LSM-800 and 100X oil immersion objective.

### RNA Co-immunoprecipitation (RIP)

Nuclei were isolated as described above, lysed in IP Lysis Buffer (150 mM KCl, 25 mM Tris-HCl ph7.5, 2 mM EDTA, 1 mM NaF, 0.5% NP-40, 0.5 mM DTT, 0.5 mM AEBSF) by vortexing and the lysate cleared by centrifugation at 16,000 × *g* for 10 min, 4°C. Co-immunoprecipitation (co-IP) of Ago1 and Ago2 was carried out as described by Beitzinger et al. (2007) and Rüdel et al. (2008), respectively, using antibodies obtained from that group. Briefly, protein G-sepharose beads were prepared by washing with IP Lysis Buffer. Beads were then coupled to 10 μg of Ago1 or Ago2 antibody in IP Lysis Buffer for 2 h, 4°C under constant rotation. Excess antibody was removed by washing twice with IP Lysis Buffer. Cleared lysate was incubated with coupled beads for 3 h, 4°C under constant rotation. Beads were then pelleted and washed 3x with IP Wash Buffer (300 mM NaCl, 50 mM Tris pH7.5, 1 mM NaF, 0.01% NP-40, 5 mM MgCl_2_) with RNase inhibitor before digestion with proteinase K for 1 h at 42°C. Finally, RNA was extracted using PCIAA with overnight precipitation at -30°C and glycogen co-precipitant.

### RNA-Seq

RNA yields from nuclear RIP were very low, and samples were sent unquantified, along with whole nucleus controls, to Beijing Genomics Institute for RNA-Seq. RIP samples were amplified using the SMARTer method before the standard Illumina library prep. Paired-end reads were aligned to the genome using TopHat (v2.0.11) (Kim et al., 2013) and counted using HTSeq (v0.6.1) (Anders et al., 2015). Genes with low read count (<10) were filtered out to minimize false positives resulting from amplification. Remaining genes were scanned for miRNA target sites against the TargetScan database (Release 6.1). Downstream analyses were performed in Excel, and final gene lists underwent functional analysis using the GATHER (Chang and Nevins, 2006) the DAVID FAC tool (Huang et al., 2007).

## Author Contributions

BG and MC designed the study and co-wrote the manuscript. BG carried out the RNA-related and IF imaging experiments and analysis. CF performed protein-related experiments and analysis. JW provided methodological support and critical appraisal of the manuscript. JA provided bioinformatic support. DW carried out and/or supervised IF imaging, and assisted with critical interpretation of nuclear localization data. MC supervised the project.

## Conflict of Interest Statement

The authors declare that the research was conducted in the absence of any commercial or financial relationships that could be construed as a potential conflict of interest.
