# Supplementary material for: miRNA Enriched in Human Neuroblast Nuclei Bind the MAZ Transcription Factor and Their Precursors Contain the MAZ Consensus Motif
Source: Front Mol Neurosci. 2017 Aug 21;10:259. doi: 10.3389/fnmol.2017.00259 (PMC5573442; doi:10.3389/fnmol.2017.00259)
Supplement: Supplementary file 1 [file Table_1.PDF]

Supplementary Table S1. Nuclear miRNA expression in SH-SY5Y cells.

Mature miRNA	Raw Expression Cytoplasm	Raw Expression Nucleus	Nuclear % of total
hsa-miR-768-5p	632.4299	10223.281	94.1742194
hsa-miR-768-3p	380.70798	5669.1206	93.70712781
hsa-miR-1299	6.865166	93.65542	93.170388
hsa-miR-297	7.3870163	94.01574	92.71517208
hsa-miR-1201	5.3476424	66.47131	92.55399554
hsa-miR-663b	45.643597	537.6604	92.17498984
hsa-miR-647	9.583795	79.09322	89.19247
hsa-miR-1248	3.388134	25.204283	88.15023578
hsa-miR-595	19.243858	142.33234	88.0899178
hsa-miR-148b*	4.166365	27.230688	86.73007623
hsa-miR-921	30.8864	160.05347	83.82401748
hsa-miR-32*	5.997028	28.369547	82.54982348
hsa-miR-593*	35.417072	147.32124	80.61869369
hsa-miR-125b-1*	7.8984885	31.823034	80.11534301
hsa-miR-1291	9.81671	36.088696	78.61535088
hsa-miR-1183	55.099842	198.55347	78.27749949
hsa-miR-300	4.4833255	16.116777	78.23639227
hsa-miR-1305	3.3115141	11.634234	77.84310241
hsa-miR-34b	5.869924	20.129953	77.42326243
hsa-miR-664*	165.62343	508.84592	75.44389082
hsa-miR-335*	4.313956	13.230105	75.41073301
hsa-miR-1279	5.3603215	15.512283	74.31886615
hsa-miR-1275	367.72522	1039.6671	73.87187533
hsa-miR-1322	4.12467	11.567449	73.71502217
hsa-miR-648	9.128713	23.488218	72.01234843
hsa-miR-574-5p	289.71848	742.6009	71.93518928
hsa-miR-554	4.4645925	11.400543	71.85909632
hsa-miR-584	7.407421	18.506092	71.41483287
hsa-miR-206	7.712482	18.785023	70.89355394
hsa-miR-640	6.592426	15.7988	70.55799446
hsa-miR-650	14.3974085	33.674038	70.04997863
hsa-miR-34c-3p	16.528984	34.26302	67.45750768
hsa-miR-601	6.52156	13.159968	66.86456458
hsa-miR-513a-5p	13.817599	27.282816	66.38087718
hsa-miR-99a*	6.326794	12.380191	66.17950995
hsa-miR-612	9.381312	18.279535	66.08450927
hsa-miR-622	3.218372	6.266047	66.06674589
hsa-miR-1273	8.719127	16.911835	65.98205327
hsa-miR-610	4.3348923	7.9906883	64.83011681
hsa-miR-564	30.666649	56.267475	64.72426754
hsa-miR-187*	30.398596	54.79749	64.31925758
hsa-miR-1306	31.652609	56.807774	64.21832246
hsa-miR-567	3.6247628	6.469873	64.09218845
hsa-miR-519e*	16.60502	29.345072	63.86292328
hsa-miR-221	55.121487	96.3623	63.61228611
hsa-miR-548l	3.1469505	5.495529	63.58741146
hsa-miR-453	3.1778023	5.505877	63.40488645

hsa-miR-31*	3.7469432	6.4586463	63.285382
hsa-miR-661	4.480894	7.623651	62.98172298
hsa-miR-608	13.722169	22.91511	62.54588393
hsa-miR-1282	2.6801555	4.386671	62.07412903
hsa-miR-630	4.6057277	7.5185857	62.01246579
hsa-miR-890	4.0902286	6.5216846	61.45625654
hsa-miR-1207-5p	2468.3574	3914.0564	61.32564454
hsa-miR-525-5p	4.190682	6.6362367	61.29386286
hsa-miR-765	38.466484	60.401913	61.093246
hsa-miR-324-5p	1886.2516	2914.5996	60.71005908
hsa-miR-337-3p	9.185412	14.087007	60.53091
hsa-miR-518d-3p	2.522268	3.8646328	60.50873375
hsa-miR-548c-5p	3.9660928	6.050573	60.40506013
hsa-miR-708*	6.542236	9.964146	60.36541503
hsa-miR-101*	7.586657	11.477868	60.20537097
hsa-miR-551b	48.60094	72.86701	59.98867191
hsa-miR-16-1*	9.591605	14.336322	59.91460104
hsa-miR-623	10.543012	15.634034	59.72421029
hsa-miR-33a*	3.1147358	4.517444	59.18943367
hsa-miR-192*	5.318157	7.6770988	59.07616532
hsa-miR-574-3p	1809.9048	2608.1238	59.03365587
hsa-miR-559	3.8197925	5.4907017	58.97325729
hsa-miR-452*	3.2060583	4.556457	58.69820315
hsa-miR-892a	3.30435	4.6420264	58.41689553
hsa-miR-583	4.011557	5.628515	58.38664898
hsa-miR-520c-5p	4.3596997	6.1051383	58.33953951
hsa-miR-582-3p	3.296154	4.5728936	58.11241503
hsa-miR-1234	28.34774	39.325138	58.11063333
hsa-miR-194*	14.054751	19.485975	58.0964616
hsa-miR-1204	2.917802	4.0212646	57.95108812
hsa-miR-1268	1735.3385	2385.5664	57.8893825
hsa-miR-211	5.611794	7.697965	57.83699765
hsa-miR-523	3.3382447	4.5559754	57.71279927
hsa-miR-1257	3.4385707	4.683028	57.661406
hsa-miR-1255b	6.1577396	8.332136	57.50315758
hsa-miR-520d-3p	3.4191885	4.6262474	57.50151337
hsa-miR-520d-5p	3.1700528	4.2831607	57.46730186
hsa-miR-1203	8.64974	11.571727	57.22496296
hsa-miR-122	2.921875	3.8751326	57.0123329
hsa-miR-659	41.95262	55.57088	56.98204023
hsa-miR-223	4.9931083	6.605541	56.95095031
hsa-miR-1289	3.2395904	4.2801175	56.91866701
hsa-miR-545	2.8832896	3.796396	56.83495044
hsa-miR-1182	39.060856	51.38503	56.81300972
hsa-miR-1246	182.69478	240.2253	56.80158294
hsa-miR-1200	3.1585228	4.1377378	56.71038943
hsa-miR-582-5p	2.8488748	3.7186584	56.62184395
hsa-miR-548d-5p	3.410385	4.438947	56.55190786
hsa-miR-28-3p	2.3852904	3.1044319	56.5498896

hsa-miR-657	4.274651	5.5456696	56.47137019
hsa-miR-519e	3.3373387	4.32068	56.42033755
hsa-miR-34c-5p	2.5835934	3.3444107	56.41714553
hsa-miR-613	3.436903	4.415497	56.23117773
hsa-miR-619	3.7575119	4.823458	56.21110499
hsa-miR-938	5.9119854	7.5669723	56.13915014
hsa-miR-371-3p	3.889109	4.9764905	56.13258866
hsa-miR-518d-5p	3.2682116	4.149583	55.94092616
hsa-miR-942	2.5239007	3.203126	55.92999942
hsa-miR-373	2.5912197	3.2747746	55.82641974
hsa-miR-631	7.3690605	9.291798	55.77022337
hsa-miR-1264	3.1065953	3.8910236	55.60496585
hsa-miR-1249	9.356734	11.693383	55.55020431
hsa-miR-518c	2.733483	3.408376	55.49420786
hsa-miR-195*	73.34733	91.03884	55.38108224
hsa-miR-219-2-3p	4.0832343	5.0679097	55.38006724
hsa-miR-662	5.065678	6.2644134	55.29005176
hsa-miR-200a	2.9892318	3.6700394	55.11172754
hsa-miR-105*	3.1712615	3.883487	55.04784472
hsa-miR-1298	3.2394285	3.9581263	54.9926525
hsa-miR-31	85.92651	104.12655	54.78814706
hsa-miR-513c	2.637334	3.1937132	54.7708343
hsa-miR-1197	2.6786785	3.2347877	54.70205782
hsa-miR-517*	3.728997	4.4925	54.6433332
hsa-miR-367*	3.4134655	4.109936	54.62869421
hsa-miR-770-5p	3.6922958	4.436167	54.57571879
hsa-miR-219-5p	2.6098216	3.1167853	54.42638816
hsa-miR-220c	5.7874875	6.8990893	54.38101553
hsa-miR-21*	9.199001	10.964189	54.3772538
hsa-miR-590-5p	24.69986	29.345154	54.29761569
hsa-miR-1258	3.8264787	4.527631	54.19645136
hsa-miR-625*	5.610435	6.636768	54.19007099
hsa-miR-620	2.9156625	3.4464567	54.1715204
hsa-miR-611	3.6524668	4.3074503	54.11426081
hsa-miR-135a	2.570999	3.0285199	54.08535901
hsa-miR-384	2.385206	2.7937984	53.94470026
hsa-miR-509-3p	3.5192115	4.1074696	53.8565799
hsa-miR-188-5p	139.63348	162.67561	53.81102169
hsa-miR-1253	3.9888973	4.6460595	53.80524313
hsa-miR-548j	3.1183698	3.6224606	53.73908532
hsa-miR-556-3p	2.6174233	3.0319834	53.66905873
hsa-miR-651	2.8599033	3.312765	53.66828151
hsa-miR-888	2.7373154	3.1691906	53.65592789
hsa-miR-1297	2.3681152	2.7233057	53.48812745
hsa-miR-639	6.2302403	7.164414	53.48711389
hsa-miR-548n	2.8945506	3.3177521	53.40615646
hsa-miR-607	2.862131	3.2787774	53.3923841
hsa-miR-224	3.6241558	4.1475096	53.36706338
hsa-let-7f-1*	6.5078316	7.430555	53.31000792

hsa-miR-431*	4.596212	5.231693	53.23304407
hsa-miR-593	4.870609	5.5384965	53.20818873
hsa-miR-200a*	3.1143503	3.539381	53.19392744
hsa-miR-542-5p	30.794899	34.946175	53.1572925
hsa-miR-454*	4.392967	4.963557	53.04915586
hsa-miR-1272	7.49785	8.471614	53.04883119
hsa-miR-558	2.6641395	3.0009592	52.97276109
hsa-miR-1207-3p	3.2574425	3.6652577	52.94549228
hsa-miR-571	3.1748924	3.570325	52.93120723
hsa-miR-875-5p	2.8811414	3.2312949	52.86427116
hsa-miR-645	3.1614268	3.5426452	52.84318546
hsa-miR-20a	22147.1	24815.264	52.84074711
hsa-miR-548e	2.8982785	3.2436092	52.81127494
hsa-miR-380	3.1891062	3.5633698	52.77130641
hsa-miR-516a-5p	2.9188411	3.2564204	52.73331988
hsa-miR-649	3.335304	3.7208922	52.73226671
hsa-miR-127-5p	4.435023	4.9468493	52.72774071
hsa-miR-548a-5p	2.6069486	2.9074504	52.72470128
hsa-miR-520b	2.9625893	3.292999	52.64091628
hsa-miR-216b	3.7474804	4.147573	52.5338182
hsa-miR-524-5p	4.482926	4.9397435	52.4240344
hsa-miR-616*	3.4697669	3.8229425	52.42142927
hsa-miR-142-3p	2.8239126	3.108936	52.40207883
hsa-miR-576-3p	3.3360138	3.6620731	52.32963169
hsa-miR-1263	8.8666315	9.71306	52.27783249
hsa-miR-1283	3.0169086	3.2998774	52.23981626
hsa-miR-573	3.7883189	4.139318	52.21376877
hsa-miR-802	2.896886	3.1621857	52.18927678
hsa-miR-325	3.5479076	3.8726857	52.18835669
hsa-miR-518e	3.7743251	4.118309	52.17914511
hsa-miR-875-3p	3.4712346	3.7845821	52.15928484
hsa-miR-493	5.715385	6.213001	52.08584799
hsa-miR-136*	2.5562398	2.767109	51.98060664
hsa-miR-147b	3.6865857	3.9817035	51.92427406
hsa-miR-568	3.016991	3.2580004	51.92039626
hsa-let-7a*	2.9226923	3.1536725	51.90064461
hsa-miR-618	3.7010784	3.9824693	51.83112613
hsa-miR-19b-2*	2.9883366	3.210801	51.79431733
hsa-miR-1259	4.0792994	4.3824115	51.79108045
hsa-miR-1256	4.790755	5.1463256	51.78910997
hsa-miR-150	5.0852675	5.4555054	51.75621799
hsa-miR-520e	3.3477771	3.5858955	51.71711598
hsa-miR-923	9101.707	9734.614	51.68001756
hsa-miR-221*	4.0695853	4.3413005	51.61525912
hsa-miR-33a	3.3727195	3.5978107	51.61459167
hsa-miR-519c-3p	3.7692604	4.01959	51.60697399
hsa-miR-132*	18.078497	19.270647	51.59595358
hsa-miR-520c-3p	3.421252	3.630685	51.48493244
hsa-miR-1284	6.09176	6.463759	51.48141626

hsa-miR-200b	3.5025787	3.7157977	51.476918
hsa-miR-183*	4.767676	5.040652	51.39155216
hsa-miR-519b-5p	2.8489125	3.0042865	51.32725711
hsa-miR-219-1-3p	9.018294	9.504196	51.3116541
hsa-miR-323-5p	14.265897	14.972653	51.20860303
hsa-miR-17*	1137.8087	1193.6232	51.19700044
hsa-miR-635	6.7392044	7.0447564	51.10836067
hsa-miR-924	2.5877779	2.6931963	50.99809615
hsa-miR-524-3p	2.6782057	2.7809374	50.94091415
hsa-miR-1265	2.7921307	2.8870814	50.83594959
hsa-miR-96	3.5496995	3.6691716	50.8274985
hsa-miR-17	28200.74	28995.975	50.69517541
hsa-miR-130a*	3.7931728	3.898547	50.68498465
hsa-miR-208b	3.6553793	3.751401	50.64820135
hsa-miR-433	4.162376	4.2701726	50.63916975
hsa-miR-200b*	5.7268033	5.864204	50.59270388
hsa-miR-220a	3.632107	3.7149916	50.56406348
hsa-miR-302a*	3.9422448	4.0262766	50.52727348
hsa-miR-621	5.052355	5.1588864	50.52163785
hsa-miR-1267	10.576594	10.79539	50.51187573
hsa-miR-106a	26101.924	26631.123	50.50177169
hsa-miR-526b	4.445262	4.527101	50.45606157
hsa-miR-371-5p	41.443954	42.179478	50.43978343
hsa-miR-130a	4143.551	4214.8184	50.42632358
hsa-miR-1321	8.454694	8.584232	50.38012372
hsa-miR-155	3.2222548	3.262654	50.31148626
hsa-miR-526b*	2.7157676	2.7452452	50.26989133
hsa-miR-302d	3.0181458	3.0461304	50.23073322
hsa-miR-519a*	2.607608	2.6296413	50.21035183
hsa-miR-106b	23379.49	23562.635	50.19507532
hsa-miR-302d*	3.795952	3.8203924	50.16044705
hsa-miR-548b-5p	3.2936203	3.310623	50.12872557
hsa-miR-217	2.812524	2.8244598	50.10587045
hsa-miR-1826	15385.52	15449.406	50.10359357
hsa-miR-450b-5p	4.667299	4.678017	50.05734424
hsa-miR-302a	2.6088722	2.6127436	50.0370709
hsa-miR-141	2.9668043	2.948818	49.84797626
hsa-miR-877*	16.881926	16.77402	49.83969252
hsa-miR-93	27809.85	27627.72	49.83573414
hsa-miR-492	4.9874964	4.9536586	49.82980951
hsa-miR-190	2.7018137	2.6827343	49.82283193
hsa-miR-617	3.650381	3.6234455	49.81484642
hsa-miR-577	2.8833935	2.8587215	49.78516627
hsa-miR-518f*	3.2712584	3.242691	49.78072135
hsa-miR-526a	5.3062987	5.2503085	49.73480968
hsa-miR-92a	27539.588	27213.69	49.70239407
hsa-miR-373*	17.401028	17.162962	49.65561557
hsa-miR-517a	3.0697553	3.016674	49.56393727
hsa-miR-521	3.8164062	3.7477138	49.54593264

hsa-miR-516b*	2.8252592	2.7735033	49.53779161
hsa-miR-222	75.8617	74.404144	49.51500755
hsa-miR-548m	3.2913883	3.2252429	49.49248777
hsa-miR-107	22732.686	22172.178	49.37589389
hsa-miR-499-3p	4.1370482	4.0244765	49.31035129
hsa-miR-191	17980.83	17451.248	49.25268001
hsa-miR-522	3.5907679	3.4843795	49.24815418
hsa-miR-552	5.5400066	5.36936	49.21788952
hsa-miR-16	21772.672	21100.967	49.21664569
hsa-let-7a	27118.63	26278.791	49.21359592
hsa-miR-26a	19085.176	18417.084	49.10926435
hsa-miR-203	10.358785	9.994086	49.10406006
hsa-miR-888*	3.0911977	2.9803555	49.08720062
hsa-miR-513a-3p	3.5532658	3.4163523	49.01778334
hsa-miR-1251	3.2913492	3.162255	48.99982865
hsa-miR-1266	17.90788	17.170723	48.94927828
hsa-miR-223*	2.807967	2.6861722	48.89159343
hsa-miR-496	3.714285	3.5516255	48.88066678
hsa-miR-141*	3.9366086	3.76356	48.8763324
hsa-miR-127-3p	7.0982704	6.785166	48.87238148
hsa-miR-103	25237.303	24046.85	48.7922558
hsa-miR-541*	10.906415	10.368959	48.73690587
hsa-miR-1293	3.0283318	2.8747005	48.69870863
hsa-miR-624*	3.2986565	3.1285775	48.67688807
hsa-miR-633	3.6889808	3.4912815	48.62331422
hsa-miR-205	3.1871598	3.0086021	48.55903355
hsa-miR-450a	2.9657066	2.7947636	48.51624091
hsa-miR-340*	3.4034946	3.2063065	48.50836586
hsa-miR-511	2.7642157	2.603205	48.50011105
hsa-miR-518a-3p	3.3465164	3.1428173	48.43050836
hsa-miR-92a-2*	5.1606674	4.8408017	48.40090642
hsa-miR-512-5p	4.0303006	3.778115	48.38516792
hsa-miR-581	3.0627563	2.8706045	48.38075075
hsa-miR-135b	3.075987	2.8821828	48.37362641
hsa-miR-646	3.1531422	2.9541428	48.37080307
hsa-miR-934	3.8929617	3.6460524	48.36245631
hsa-miR-544	3.5599186	3.3303945	48.33444361
hsa-miR-376b	4.2792397	4.0026875	48.3303874
hsa-miR-580	3.8000863	3.549648	48.29627651
hsa-miR-25	13719.228	12774.609	48.2172854
hsa-miR-548k	3.3749483	3.139725	48.19466542
hsa-miR-590-3p	2.784127	2.5872595	48.16744243
hsa-miR-510	7.707245	7.1607804	48.16228253
hsa-miR-18a	10299.919	9552.46	48.11745736
hsa-miR-497*	3.397333	3.143677	48.06103339
hsa-let-7i*	31.507704	29.127253	48.03706383
hsa-miR-655	3.7595193	3.4735744	48.02335687
hsa-miR-23b	26421.889	24411.932	48.02301208
hsa-miR-302f	3.1396685	2.898997	48.00724597

hsa-miR-886-5p	4.399567	4.0422225	47.88347897
hsa-miR-578	2.8769958	2.6390364	47.84302021
hsa-miR-20b	10304.625	9439.018	47.80788429
hsa-miR-515-3p	2.8723247	2.62736	47.77292051
hsa-miR-586	3.3801386	3.0867043	47.73123992
hsa-miR-374a*	5.259968	4.8000264	47.71400668
hsa-miR-587	3.1764057	2.897768	47.70637363
hsa-miR-668	17.36867	15.834969	47.69046248
hsa-miR-200c*	3.9052255	3.559974	47.68759361
hsa-miR-450b-3p	2.993779	2.7275324	47.67320304
hsa-miR-1245	3.9821618	3.6279736	47.67291788
hsa-miR-512-3p	4.260385	3.878906	47.65655878
hsa-miR-144*	5.374865	4.8916645	47.64671937
hsa-miR-519a	3.2064798	2.916232	47.62974472
hsa-miR-190b	3.093375	2.8103218	47.60274613
hsa-miR-29a*	3.1420438	2.8477147	47.54306371
hsa-miR-493*	3.500108	3.1667125	47.49959145
hsa-miR-100*	3.6103735	3.2580488	47.43518464
hsa-miR-624	3.175623	2.8565586	47.35531503
hsa-miR-146a*	3.4873874	3.1357453	47.34534913
hsa-miR-185*	56.465794	50.716785	47.31812341
hsa-miR-302e	3.3850715	3.0356362	47.27884124
hsa-miR-513b	3.7666438	3.3743303	47.25308134
hsa-miR-33b	2.8897297	2.5874913	47.2409512
hsa-miR-24	23655.766	21179.133	47.23805221
hsa-miR-886-3p	8.870412	7.9264417	47.19003833
hsa-miR-506	3.0699904	2.7366095	47.12929334
hsa-miR-130b	10085.375	8957.337	47.03813721
hsa-miR-23a	18273.408	16211.88	47.01100365
hsa-miR-181a	7701.815	6827.775	46.99220694
hsa-miR-136	3.3223932	2.945058	46.98972367
hsa-miR-891b	3.030986	2.6855495	46.97862018
hsa-miR-1205	2.8775558	2.5486643	46.96942352
hsa-miR-525-3p	3.0113728	2.665569	46.95431262
hsa-miR-218-1*	3.5492313	3.1372058	46.91894582
hsa-miR-27b	12020.3125	10614.913	46.89554783
hsa-miR-632	5.237619	4.6251435	46.8950104
hsa-miR-302b*	3.8714244	3.4181817	46.89117153
hsa-miR-144	2.6514537	2.3325925	46.80118134
hsa-miR-142-5p	3.3428752	2.9347134	46.74905584
hsa-miR-519d	3.0182552	2.6477396	46.73035704
hsa-miR-1	3.569641	3.1311638	46.7281751
hsa-let-7b	23489.084	20566.055	46.68253345
hsa-miR-671-3p	51.56505	44.857098	46.52157096
hsa-miR-124	9663.6045	8397.491	46.494915
hsa-miR-1185	4.2818704	3.7096741	46.41998928
hsa-miR-1261	3.606088	3.1119373	46.32220275
hsa-miR-1302	4.1243834	3.5589752	46.32056611
hsa-miR-508-5p	10.589528	9.114369	46.25668212

hsa-miR-1276	16.997414	14.627948	46.25385158
hsa-miR-1286	8.19825	7.0539894	46.24887674
hsa-miR-196b	3.353296	2.8835971	46.23451218
hsa-miR-208a	3.848818	3.3073132	46.21649754
hsa-miR-15b*	3.9764357	3.411403	46.17592693
hsa-miR-597	3.5997307	3.08338	46.1368985
hsa-miR-585	3.5098183	2.9989555	46.07558339
hsa-miR-548d-3p	3.4418309	2.940689	46.07410625
hsa-miR-29b	21.558142	18.409243	46.06066421
hsa-miR-892b	3.4537413	2.9483716	46.05310225
hsa-miR-298	17.684969	15.095778	46.05074436
hsa-miR-27a	6704.2554	5721.891	46.04718805
hsa-let-7f-2*	3.148966	2.6872451	46.04434374
hsa-miR-599	2.9292924	2.4978106	46.02475022
hsa-miR-220b	4.2173824	3.5933197	46.00507936
hsa-miR-101	24.00448	20.431744	45.97992845
hsa-miR-548a-3p	8.468829	7.2083206	45.97979087
hsa-miR-579	3.4952424	2.953388	45.79868618
hsa-miR-138	3600.6326	3038.1096	45.76333149
hsa-miR-19b	16652.049	14046.669	45.75653289
hsa-miR-125a-5p	13925.739	11730.472	45.72176305
hsa-miR-609	4.5577216	3.8305705	45.66567848
hsa-miR-518c*	7.3899	6.1926637	45.5927455
hsa-miR-555	3.56691	2.9767673	45.49074112
hsa-miR-449a	3.3514416	2.7890415	45.42055494
hsa-let-7c	20489.117	17014.72	45.36794462
hsa-miR-199a-3p	10460.374	8681.306	45.35289483
hsa-miR-643	8.299049	6.8839583	45.33988665
hsa-miR-26a-2*	4.1422777	3.4331005	45.31919608
hsa-miR-181a*	7.5571294	6.2602882	45.30722296
hsa-miR-199b-3p	10427.594	8636.328	45.30194784
hsa-miR-520f	3.3676414	2.7873814	45.28628879
hsa-miR-641	60.43385	49.994785	45.27338856
hsa-miR-30d*	4.6543574	3.8499382	45.27051247
hsa-miR-542-3p	4.448405	3.6746228	45.23710728
hsa-miR-576-5p	4.164822	3.4384086	45.22299508
hsa-miR-15b	12492.32	10306.829	45.20707769
hsa-miR-377	4.003966	3.3015594	45.19263461
hsa-miR-345	4294.2495	3535.5667	45.15516852
hsa-miR-606	5.589599	4.5983396	45.13513264
hsa-miR-372	4.8431277	3.9718914	45.05822795
hsa-miR-889	3.4919436	2.862881	45.0505117
hsa-miR-30e	249.34102	204.28271	45.03351489
hsa-miR-876-5p	3.9035428	3.1947062	45.00696158
hsa-miR-182*	3.7450988	3.0599258	44.96568315
hsa-miR-431	3.3888009	2.767707	44.95579385
hsa-miR-600	3.8845527	3.1661298	44.90529534
hsa-miR-1324	4.6002517	3.7447014	44.87384597
hsa-miR-96*	3.423793	2.7857406	44.86231623

hsa-miR-34b*	10.033456	8.162114	44.85769888
hsa-miR-515-5p	3.6291256	2.950598	44.84379861
hsa-miR-561	3.523814	2.864322	44.83814997
hsa-miR-448	3.2552357	2.644411	44.8232095
hsa-miR-147	2.9908972	2.4296367	44.82283009
hsa-miR-634	9.737343	7.90015	44.79179666
hsa-miR-556-5p	3.7645156	3.0525658	44.77819203
hsa-miR-626	3.6242237	2.9331799	44.73081236
hsa-miR-920	8.592895	6.94873	44.71044694
hsa-miR-1208	28.558182	23.085054	44.70102145
hsa-miR-452	4.295917	3.4642384	44.64135345
hsa-miR-664	9.899732	7.9809494	44.63448132
hsa-miR-378*	8.251946	6.647589	44.61608366
hsa-miR-520a-5p	3.0901012	2.4892614	44.61551576
hsa-miR-361-5p	11465.151	9235.22	44.61378977
hsa-miR-202*	5.447723	4.385235	44.59731243
hsa-miR-1237	18.096575	14.507001	44.49512225
hsa-let-7i	8590.974	6881.6504	44.47629712
hsa-miR-143	4757.453	3807.661	44.45546201
hsa-miR-604	4.137121	3.3058746	44.41591501
hsa-let-7d	14033.391	11195.368	44.37542092
hsa-miR-514	3.6575105	2.90911	44.30147897
hsa-miR-517b	3.1241486	2.482011	44.27292794
hsa-miR-181d	2749.0999	2177.0361	44.19358499
hsa-miR-591	4.340592	3.4346695	44.17432777
hsa-miR-1227	6.9475174	5.476904	44.08176303
hsa-miR-519b-3p	6.2724304	4.9345155	44.0308675
hsa-miR-199a-5p	5071.175	3977.961	43.95956697
hsa-miR-548c-3p	4.20713	3.3001058	43.9590002
hsa-miR-1252	3.5729873	2.8002408	43.93755811
hsa-miR-451	4.513478	3.527753	43.87080784
hsa-miR-499-5p	4.6911893	3.6601017	43.8267772
hsa-miR-549	3.7694595	2.9362898	43.78764652
hsa-miR-518f	4.570228	3.5558262	43.75833723
hsa-miR-1179	3.5373437	2.7514098	43.7512744
hsa-miR-412	6.9167075	5.360126	43.66049275
hsa-miR-644	3.6553924	2.8325608	43.65877362
hsa-miR-509-3-5p	4.9035316	3.7837975	43.55536042
hsa-miR-523*	4.517999	3.478803	43.5024276
hsa-miR-1290	13.778359	10.607621	43.49885057
hsa-miR-30c	6574.3438	5060.5747	43.4947155
hsa-miR-29b-1*	18.79127	14.46062	43.48811451
hsa-miR-516b	3.4656603	2.6666784	43.48550415
hsa-miR-302b	3.3438606	2.5709386	43.46620254
hsa-miR-449b	3.9528627	3.038867	43.46373688
hsa-miR-656	4.3375473	3.3245306	43.38941268
hsa-miR-545*	5.1539326	3.9399767	43.32544531
hsa-miR-1827	4.7210183	3.596571	43.24054567
hsa-miR-181b	6536.9434	4971.7007	43.19970847

hsa-miR-342-3p	9746.556	7406.7847	43.17983785
hsa-miR-149*	4853.676	3687.5615	43.17362092
hsa-miR-99b	10797.437	8201.2	43.16730721
hsa-miR-1181	49.17155	37.338676	43.16099694
hsa-miR-548g	4.894737	3.7165768	43.15923082
hsa-miR-548h	4.3678203	3.31621	43.15716975
hsa-miR-548f	3.6826026	2.7945817	43.14500824
hsa-let-7g*	37.81726	28.665335	43.11705191
hsa-miR-548i	4.361241	3.3044393	43.10692816
hsa-miR-215	4.308076	3.2601032	43.07645358
hsa-miR-665	191.99348	144.76271	42.98739394
hsa-miR-1308	9893.656	7452.6387	42.96386536
hsa-miR-1206	4.2217097	3.1532876	42.75645769
hsa-miR-411*	21.586021	16.08784	42.70292339
hsa-miR-520h	3.886547	2.8934937	42.67664204
hsa-miR-34a	2708.0789	2016.1216	42.67646134
hsa-miR-222*	4.5615673	3.395012	42.66924104
hsa-miR-106a*	3.3045971	2.457721	42.65160231
hsa-miR-145	7395.7036	5485.266	42.58426322
hsa-miR-148a*	7.0693603	5.2428803	42.58266607
hsa-miR-566	5.7547507	4.257769	42.52445066
hsa-miR-519c-5p	4.242604	3.1349556	42.49312469
hsa-miR-126*	3.8267748	2.825554	42.4746594
hsa-miR-153	195.0334	143.96571	42.46787256
hsa-miR-1274a	5.6004386	4.1293583	42.44033398
hsa-miR-7-2*	9.71978	7.1630273	42.42793969
hsa-miR-548o	3.3828638	2.4926972	42.42483739
hsa-miR-15a*	38.331287	28.200617	42.38660748
hsa-miR-944	7.144875	5.2549014	42.37900129
hsa-miR-518a-5p	5.190266	3.8123329	42.34702603
hsa-miR-516a-3p	3.7375634	2.7441301	42.33662237
hsa-miR-936	28.011124	20.564854	42.33543996
hsa-miR-548p	3.8830745	2.8501675	42.32979447
hsa-miR-605	13.371555	9.787105	42.26110233
hsa-miR-491-3p	3.935405	2.8789237	42.24808968
hsa-miR-10a*	4.1738095	3.0484712	42.20925947
hsa-miR-522*	3.9422925	2.8774107	42.19260891
hsa-miR-429	3.6850493	2.6819248	42.12243929
hsa-miR-455-5p	4.2260685	3.0749404	42.11665048
hsa-miR-369-5p	3.9693956	2.8879874	42.11500801
hsa-miR-196a	3.6287386	2.6372669	42.08848684
hsa-miR-539	8.43481	6.1032157	41.98104905
hsa-miR-320c	14189.629	10266.234	41.97862083
hsa-miR-338-3p	15.202235	10.986487	41.95121473
hsa-miR-1294	7.2176814	5.204737	41.89793672
hsa-miR-615-5p	14.008475	10.095328	41.88271867
hsa-miR-1295	3.8764875	2.7900093	41.85120587
hsa-miR-588	5.4109206	3.890702	41.82820748
hsa-miR-1233	12.981563	9.3216	41.79496872

hsa-miR-504	5.5793343	4.0051365	41.78776881
hsa-miR-320a	15371.7295	10998.757	41.70858585
hsa-miR-425	7851.2896	5575.145	41.52364471
hsa-miR-527	5.167888	3.6574929	41.44289002
hsa-miR-1243	6.590529	4.6560936	41.39992748
hsa-miR-210	3213.9978	2270.5806	41.39936444
hsa-miR-518b	4.4984603	3.169276	41.33261599
hsa-miR-1238	35.93812	25.263243	41.27888949
hsa-miR-16-2*	13.439743	9.396085	41.14624177
hsa-miR-125b	7945.5903	5553.01	41.13767262
hsa-miR-520a-3p	4.938909	3.4505959	41.12991102
hsa-miR-488	4.5527186	3.1754606	41.08937588
hsa-miR-320b	16240.847	11228.737	40.87698234
hsa-miR-30b	3479.0618	2402.4988	40.84798174
hsa-miR-381	75.8661	52.33984	40.8248167
hsa-miR-483-5p	294.70743	203.30542	40.82332815
hsa-miR-380*	5.2414165	3.609587	40.78166956
hsa-miR-369-3p	3.904998	2.6854222	40.74735933
hsa-miR-337-5p	7.9960475	5.479612	40.66303397
hsa-miR-19a*	3.8829224	2.6577854	40.63452277
hsa-miR-155*	5.6413236	3.860359	40.62816201
hsa-miR-216a	3.9659405	2.6996136	40.500963
hsa-miR-151-5p	10537.483	7154.777	40.44015293
hsa-miR-638	12662.414	8591.204	40.42231304
hsa-miR-365	5.974645	4.0399933	40.34088081
hsa-miR-424	3.966941	2.6729705	40.25611637
hsa-miR-1178	4.1596313	2.786174	40.11304492
hsa-miR-186*	3.8625596	2.5826144	40.07051478
hsa-miR-376a*	3.7822	2.520605	39.9917973
hsa-miR-508-3p	3.8673623	2.5735362	39.95616761
hsa-miR-146a	8.069615	5.362516	39.92304721
hsa-miR-143*	37.310337	24.761805	39.89197763
hsa-miR-520g	4.305188	2.8469613	39.80567492
hsa-miR-362-3p	19.207586	12.634624	39.67885395
hsa-miR-488*	6.4201865	4.219933	39.660579
hsa-miR-1236	14.296849	9.39478	39.65442815
hsa-miR-767-3p	13.5478735	8.864188	39.55097125
hsa-miR-302c*	6.398643	4.1635556	39.4194027
hsa-miR-658	42.499714	27.622915	39.39229803
hsa-let-7g	4822.756	3134.2605	39.3898957
hsa-miR-379*	6.5605245	4.2583847	39.36057343
hsa-miR-507	3.9622052	2.5686483	39.33097412
hsa-miR-575	64.91419	42.081123	39.32987513
hsa-miR-517c	5.0982327	3.2976859	39.27724954
hsa-miR-653	4.600092	2.9532154	39.09830811
hsa-miR-553	5.1060414	3.262904	38.98823381
hsa-miR-562	4.2026696	2.676555	38.90780074
hsa-miR-122*	4.596584	2.9196393	38.8444992
hsa-miR-708	7822.0835	4967.0376	38.8379902

hsa-miR-603	8.5528965	5.430737	38.83637969
hsa-miR-302c	4.280043	2.7051468	38.72689043
hsa-miR-30a	615.6221	388.6366	38.69885319
hsa-miR-99a	4628.558	2921.4666	38.69479578
hsa-miR-374b*	5.789959	3.648667	38.65676
hsa-miR-296-3p	537.3803	338.02127	38.61328122
hsa-miR-1262	8.995952	5.6463904	38.56207051
hsa-miR-140-3p	3604.8557	2257.2546	38.50583637
hsa-miR-432	52.99394	33.11374	38.4561981
hsa-miR-518e*	4.3468566	2.7155964	38.45117837
hsa-miR-1278	5.9230056	3.6850863	38.35398681
hsa-miR-95	32.557064	20.205027	38.29459109
hsa-miR-29c*	53.275665	32.883316	38.16585992
hsa-miR-367	4.233122	2.610379	38.14391201
hsa-miR-138-2*	40.14981	24.658813	38.04866059
hsa-miR-375	3826.7937	2340.0747	37.94591595
hsa-miR-1287	9.064453	5.5342045	37.90899608
hsa-miR-320d	10423.91	6346.7954	37.84453455
hsa-miR-486-3p	64.286064	38.916634	37.70893083
hsa-miR-18b	2318.6946	1403.2552	37.70215278
hsa-miR-93*	5303.596	3207.827	37.68849228
hsa-miR-614	6.0681863	3.6696854	37.68467601
hsa-miR-193a-3p	21.666315	13.082127	37.64809657
hsa-miR-509-5p	7.4148526	4.469438	37.60794944
hsa-miR-32	5.8875847	3.5341187	37.51040072
hsa-miR-137	1112.6227	667.2181	37.48751574
hsa-miR-654-3p	174.40724	104.34808	37.43357436
hsa-miR-570	7.200482	4.306048	37.4226461
hsa-miR-1228	66.75829	39.919277	37.42049816
hsa-miR-145*	6.602522	3.9442818	37.39788731
hsa-miR-299-5p	32.621754	19.482431	37.39129784
hsa-miR-876-3p	4.4673877	2.6547158	37.27432212
hsa-miR-410	20.896404	12.234689	36.92811764
hsa-miR-15a	2452.536	1427.0962	36.78431682
hsa-miR-485-3p	98.0187	56.92505	36.73917147
hsa-miR-301a	992.2229	575.17413	36.69613499
hsa-miR-1226*	82.242546	47.48749	36.60485379
hsa-miR-23a*	89.07064	51.40034	36.59142977
hsa-miR-1228*	5564.5923	3209.8206	36.58159966
hsa-miR-569	4.1250553	2.3734403	36.52291924
hsa-miR-922	5.381914	3.0936415	36.50075207
hsa-let-7f	5297.2363	3043.2263	36.48750011
hsa-let-7b*	60.443523	34.68594	36.46182676
hsa-miR-125a-3p	419.30136	239.21082	36.32595224
hsa-miR-873	144.09044	81.96584	36.25904133
hsa-miR-1825	43.063076	24.461578	36.22614342
hsa-miR-363	1705.6382	958.2046	35.97076374
hsa-miR-146b-3p	7.4377236	4.1577992	35.85693609
hsa-miR-1224-5p	212.3891	118.50297	35.8131792

hsa-miR-154*	18.077402	10.05644	35.74499352
hsa-miR-106b*	2137.2578	1185.9603	35.68710402
hsa-miR-214	6609.267	3657.4504	35.62434084
hsa-miR-186	12.825709	7.0726643	35.54393213
hsa-miR-30d	3162.215	1741.2155	35.51014948
hsa-miR-421	3220.1897	1766.4113	35.42315297
hsa-miR-483-3p	63.781067	34.978695	35.41796202
hsa-miR-495	162.80894	89.05856	35.3592901
hsa-miR-376c	160.07329	87.44773	35.32941566
hsa-let-7e	14878.8125	8101.4453	35.25393566
hsa-miR-299-3p	51.76652	28.150625	35.2247631
hsa-miR-132	3294.2664	1789.2266	35.1967948
hsa-let-7e*	92.178856	50.035244	35.18304022
hsa-miR-10b	2523.8643	1359.8872	35.01478403
hsa-miR-432*	9.844659	5.3002605	34.99695393
hsa-miR-497	954.1976	513.4554	34.98479545
hsa-miR-1277	4.386895	2.359952	34.97859074
hsa-miR-563	7.7883124	4.153627	34.78184624
hsa-miR-1323	16.250906	8.652286	34.74368266
hsa-miR-589	54.996784	29.213501	34.69113185
hsa-miR-22*	16.666784	8.818276	34.60174706
hsa-miR-204	6.121811	3.2362518	34.58249714
hsa-miR-543	210.94055	111.30194	34.53980883
hsa-miR-423-3p	6008.9478	3162.955	34.48526515
hsa-miR-758	63.930157	33.62547	34.4679964
hsa-miR-338-5p	250.85864	130.48222	34.21669002
hsa-miR-18b*	8.195367	4.259317	34.19851519
hsa-miR-1304	14.637303	7.5628333	34.06660751
hsa-miR-128	2147.9683	1106.7109	34.00368614
hsa-miR-199b-5p	389.52896	199.8553	33.90916819
hsa-miR-642	8.610816	4.41462	33.89230119
hsa-miR-744	2459.0354	1253.9127	33.77135005
hsa-miR-769-5p	978.85205	498.99512	33.76500156
hsa-miR-7	336.09988	171.27107	33.7565779
hsa-miR-548b-3p	5.7590203	2.9271376	33.69887623
hsa-miR-933	95.66571	48.510548	33.64669653
hsa-miR-935	1852.5963	936.7695	33.58360169
hsa-miR-195	3552.36	1795.7402	33.57716073
hsa-miR-184	22.6047	11.374095	33.4740976
hsa-miR-92b	3238.6301	1625.625	33.41981386
hsa-miR-377*	20.77823	10.42332	33.40641731
hsa-miR-196a*	6.263415	3.122507	33.26798369
hsa-miR-596	46.975697	23.413834	33.26323342
hsa-miR-340	26.964125	13.397609	33.193839
hsa-miR-185	4409.517	2188.3547	33.16758494
hsa-miR-376a	60.821056	30.087952	33.09677738
hsa-miR-424*	118.152374	58.315388	33.04591577
hsa-miR-335	14.979299	7.3926606	33.0443141
hsa-miR-503	206.67104	101.64292	32.96734277

hsa-miR-126	2205.6152	1081.2194	32.89546118
hsa-miR-24-1*	14.831882	7.2496743	32.83135573
hsa-miR-1224-3p	27.974443	13.622742	32.74919204
hsa-miR-139-5p	1713.3131	832.02985	32.68832006
hsa-miR-19b-1*	59.287434	28.64018	32.57245215
hsa-miR-187	69.1202	33.06917	32.36067509
hsa-miR-744*	109.31112	52.253696	32.34224957
hsa-miR-487a	214.22939	102.29443	32.31808273
hsa-miR-652	2328.2744	1111.4795	32.31276226
hsa-miR-1260	72.61321	34.534145	32.23051563
hsa-miR-1229	48.803143	23.191439	32.21275595
hsa-miR-140-5p	402.69092	191.08788	32.18166091
hsa-miR-22	1478.6045	700.2229	32.13760301
hsa-miR-627	14.474841	6.765982	31.85367158
hsa-miR-1255a	13.76887	6.4180646	31.79316091
hsa-miR-151-3p	2723.9238	1269.1023	31.78297031
hsa-miR-592	1345.8695	625.6677	31.73501875
hsa-miR-411	109.74756	50.847046	31.66173962
hsa-miR-182	114.2431	52.822018	31.6176223
hsa-miR-532-5p	1298.3613	600.2607	31.61559805
hsa-miR-100	522.8905	240.40733	31.49587495
hsa-miR-490-3p	487.9979	224.25957	31.4857449
hsa-miR-181a-2*	2184.8474	1003.25464	31.46871171
hsa-miR-494	940.9046	427.87183	31.25943877
hsa-miR-663	3579.1963	1626.3912	31.24318245
hsa-miR-34a*	187.81563	85.01151	31.15947702
hsa-miR-214*	672.46674	302.91742	31.05621687
hsa-miR-9*	740.9289	332.93002	31.00314332
hsa-miR-551b*	85.930565	38.510906	30.94700319
hsa-miR-1184	52.97337	23.732065	30.93922223
hsa-miR-26a-1*	5.3226695	2.3803518	30.9015347
hsa-miR-885-5p	601.827	268.69217	30.86573843
hsa-miR-326	95.05505	42.315533	30.80392619
hsa-miR-19a	1403.7738	624.6968	30.79644339
hsa-miR-133b	10.487533	4.663998	30.7823546
hsa-miR-129-5p	185.12183	82.247284	30.76169972
hsa-miR-29c	80.8379	35.721928	30.6468606
hsa-miR-329	126.11017	55.58017	30.59060267
hsa-miR-541	12.303983	5.3981423	30.49431754
hsa-miR-197	2092.545	917.51416	30.48159891
hsa-miR-30c-2*	33.97245	14.822426	30.37701336
hsa-miR-625	509.4021	221.86601	30.33989955
hsa-miR-20a*	34.32558	14.933715	30.31654229
hsa-miR-152	2242.7832	975.2171	30.30506554
hsa-miR-1307	2584.8445	1123.2617	30.29205852
hsa-miR-301b	179.97856	78.12046	30.26763139
hsa-miR-1288	10.534538	4.5602584	30.21079768
hsa-miR-382	390.59628	168.70827	30.16393662
hsa-miR-1271	2490.4084	1074.1149	30.13347956

hsa-miR-192	311.59622	134.18007	30.10031556
hsa-miR-671-5p	980.2797	421.8363	30.08569191
hsa-miR-99b*	1365.4532	586.8093	30.05790973
hsa-miR-330-5p	92.52128	39.752354	30.05312003
hsa-miR-138-1*	397.49033	170.48048	30.01571155
hsa-miR-181c	813.0976	348.1464	29.98046922
hsa-miR-1250	191.3039	81.87384	29.97090466
hsa-miR-29b-2*	266.52118	114.03231	29.96485724
hsa-miR-1202	641.04987	273.59927	29.91302982
hsa-miR-339-5p	3321.1982	1416.4404	29.89760342
hsa-miR-943	99.69102	42.49708	29.88793014
hsa-miR-129*	40.08797	17.072344	29.86747763
hsa-miR-636	76.66967	32.603943	29.8369772
hsa-miR-1269	171.49512	72.76605	29.79026507
hsa-miR-324-3p	2218.2615	940.0103	29.76343898
hsa-miR-331-3p	1001.8779	424.1207	29.74201377
hsa-miR-370	264.17496	111.818184	29.73942099
hsa-miR-30e*	228.07176	96.346306	29.69819381
hsa-miR-181c*	586.7198	247.23146	29.64579249
hsa-miR-24-2*	334.3285	140.34834	29.56713456
hsa-miR-629*	318.87527	133.46513	29.50546314
hsa-miR-1226	309.7229	129.56133	29.49373575
hsa-miR-939	332.73032	139.15495	29.48914892
hsa-miR-1180	1962.0685	818.5161	29.43683497
hsa-miR-154	59.267292	24.54339	29.28432202
hsa-miR-125b-2*	499.06763	206.39946	29.25713516
hsa-miR-629	606.5737	250.68318	29.24248097
hsa-miR-1281	310.90738	128.31776	29.21457547
hsa-miR-660	327.54877	135.1198	29.20444758
hsa-miR-550	195.1991	79.6067	28.96834783
hsa-miR-766	756.5275	306.55756	28.83659752
hsa-miR-33b*	40.554043	16.41497	28.81385711
hsa-miR-484	902.0052	364.77283	28.79532336
hsa-miR-202	22.089617	8.929849	28.78788758
hsa-miR-193b*	52.8458	21.349073	28.7743238
hsa-miR-212	303.25534	122.51069	28.77418144
hsa-miR-891a	38.750557	15.635132	28.74861436
hsa-miR-502-5p	36.958496	14.888626	28.71639818
hsa-miR-29a	856.5	343.88718	28.64802172
hsa-miR-487b	1081.8497	433.3369	28.59957315
hsa-miR-30c-1*	82.18042	32.815083	28.53597066
hsa-miR-637	67.16569	26.788177	28.51205369
hsa-miR-188-3p	12.082149	4.8050065	28.45361671
hsa-miR-887	744.5825	295.98254	28.44440555
hsa-miR-218	1839.7618	731.08496	28.43751605
hsa-miR-191*	151.29471	60.037632	28.4091074
hsa-miR-149	5297.1733	2101.8694	28.40731545
hsa-miR-331-5p	293.52988	116.35721	28.38762499
hsa-miR-654-5p	28.267822	11.202819	28.38266295

hsa-miR-330-3p	1869.8148	740.3151	28.36315158
hsa-miR-7-1*	260.75085	103.06677	28.3292409
hsa-miR-628-5p	26.714376	10.548638	28.30860112
hsa-miR-362-5p	808.7078	319.12466	28.29539593
hsa-miR-339-3p	1215.1195	479.41953	28.29203232
hsa-miR-194	645.00226	254.0509	28.25760604
hsa-miR-760	113.66238	44.480946	28.12698273
hsa-miR-124*	145.80684	56.72968	28.00960538
hsa-miR-505	507.35245	196.97809	27.96671148
hsa-miR-425*	1005.52997	390.35107	27.96449402
hsa-miR-10b*	356.82495	137.25322	27.77965681
hsa-miR-490-5p	1397.1348	537.33496	27.77686016
hsa-miR-551a	131.39317	50.49712	27.76240557
hsa-miR-489	113.07363	43.427612	27.74905262
hsa-miR-328	293.80484	112.30932	27.6546181
hsa-miR-1280	387.67264	147.38832	27.54607998
hsa-miR-198	92.7539	35.22388	27.52343415
hsa-let-7d*	105.246185	39.83841	27.45874571
hsa-miR-877	813.2449	307.5566	27.44077341
hsa-miR-491-5p	464.95657	175.20142	27.36846571
hsa-miR-150*	197.69627	73.95208	27.22346004
hsa-miR-502-3p	814.3129	304.0321	27.18589523
hsa-miR-628-3p	302.96664	112.68337	27.11015693
hsa-miR-1225-3p	23.288248	8.658171	27.10216441
hsa-miR-498	40.952465	15.189921	27.05606598
hsa-miR-27b*	719.6982	266.65915	27.0347405
hsa-miR-374b	411.51248	152.36098	27.0204205
hsa-miR-30a*	218.10283	80.699524	27.00766005
hsa-miR-148a	184.0973	67.92228	26.95119165
hsa-miR-10a	10.158043	3.7315404	26.86574746
hsa-miR-105	779.78973	286.1954	26.84797301
hsa-miR-9	146.54149	53.517597	26.75089535
hsa-miR-146b-5p	254.7885	92.72709	26.68285759
hsa-miR-589*	501.18466	182.18494	26.65979581
hsa-miR-409-3p	611.6479	221.88708	26.6200082
hsa-miR-1254	95.390625	34.55958	26.59447902
hsa-miR-21	254.30336	92.10258	26.58804869
hsa-miR-27a*	123.75695	44.773777	26.5671298
hsa-miR-139-3p	91.41864	32.901733	26.46527854
hsa-miR-500*	806.8135	290.13004	26.44894923
hsa-miR-18a*	1327.5199	476.57788	26.41641076
hsa-miR-296-5p	29.223566	10.47038	26.37777559
hsa-miR-885-3p	136.9282	48.912388	26.31954006
hsa-miR-378	374.83838	133.52217	26.26525013
hsa-miR-501-3p	541.23627	192.77397	26.26311726
hsa-miR-20b*	231.12207	82.25411	26.24772247
hsa-miR-323-3p	58.38815	20.771545	26.24005183
hsa-miR-557	35.016495	12.449454	26.22817886
hsa-miR-486-5p	1249.3412	443.42178	26.19514871

hsa-miR-675	2291.3833	809.06757	26.09515854
hsa-miR-500	589.19336	207.49188	26.04439866
hsa-miR-423-5p	989.8256	345.8602	25.89382922
hsa-miR-193b	1086.9585	379.6605	25.8867845
hsa-miR-1296	532.92285	186.11589	25.88398645
hsa-miR-454	234.58423	81.6499	25.81944586
hsa-miR-129-3p	156.37105	54.205524	25.74147873
hsa-miR-422a	165.26031	57.13754	25.6915883
hsa-miR-183	16.60586	5.7205596	25.62237789
hsa-miR-501-5p	313.9296	107.47678	25.50430774
hsa-miR-616	17.757753	6.0729127	25.48360493
hsa-miR-550*	562.6246	191.15251	25.35928824
hsa-miR-769-3p	501.6558	170.2212	25.33517296
hsa-miR-940	117.03887	39.67597	25.31730243
hsa-miR-98	175.18718	59.189175	25.253902
hsa-miR-1231	329.1135	110.88386	25.20102848
hsa-miR-615-3p	379.44247	127.822754	25.19840666
hsa-miR-455-3p	357.856	119.92273	25.10005625
hsa-miR-134	264.4685	88.40867	25.05366669
hsa-miR-1247	73.05303	24.204203	24.88678965
hsa-miR-148b	493.11063	163.29478	24.87712281
hsa-miR-200c	61.29306	20.254953	24.83807055
hsa-miR-218-2*	540.53925	176.72583	24.63884482
hsa-miR-130b*	164.5981	53.63681	24.57755728
hsa-miR-135a*	392.41373	124.69599	24.11402942
hsa-miR-92b*	383.83472	121.76681	24.08355252
hsa-miR-874	614.94147	193.81795	23.96484606
hsa-miR-379	486.32065	152.74449	23.90123955
hsa-miR-941	344.6631	108.20435	23.89316123
hsa-miR-767-5p	668.34955	208.60313	23.78727322
hsa-miR-133a	15.286804	4.722302	23.60076457
hsa-miR-346	767.073	234.57372	23.41880778
hsa-miR-1225-5p	736.13544	225.11067	23.41863001
hsa-miR-409-5p	111.967384	34.163166	23.3785242
hsa-miR-92a-1*	286.09787	85.921265	23.09592624
hsa-miR-1303	171.91893	51.587677	23.08105236
hsa-miR-361-3p	39.726677	11.8517065	22.97804952
hsa-miR-26b	163.5984	48.732555	22.95122489
hsa-miR-26b*	18.657608	5.503636	22.77877745
hsa-miR-720	167.36076	49.14256	22.69829396
hsa-miR-532-3p	682.23413	197.67317	22.46522673
hsa-miR-135b*	17.927763	5.17342	22.3946107
hsa-miR-937	21.20896	6.110401	22.36655901
hsa-miR-1301	2243.9705	640.3619	22.20139052
hsa-miR-23b*	372.55435	104.15783	21.84920679
hsa-miR-1270	333.5906	92.33719	21.67907147
hsa-miR-1292	130.29216	35.05202	21.19942776
hsa-miR-193a-5p	170.92229	45.8644	21.15646491
hsa-miR-505*	1158.1102	304.45657	20.81659287

hsa-miR-1285	331.3991	87.08072	20.8088218
hsa-miR-28-5p	37.09463	9.643445	20.6329529
hsa-miR-485-5p	65.90208	17.015638	20.52111227
hsa-let-7c*	27.7213	7.1033173	20.39740233
hsa-miR-1244	363.41016	92.60647	20.3076958
hsa-miR-1274b	25.604078	6.467405	20.16559384
hsa-miR-342-5p	617.91864	153.23999	19.87139663
hsa-miR-25*	1114.6824	273.0684	19.67704865
hsa-miR-383	25.511606	6.1408715	19.40091893
hsa-miR-1300	553.7117	130.3384	19.05392602
hsa-miR-374a	27.848215	5.8543243	17.37057332
hsa-miR-602	152.74074	30.534098	16.66027826
hsa-miR-572	340.56937	67.493095	16.53989298
hsa-miR-30b*	212.2274	40.460518	16.01205088
hsa-miR-598	321.70288	57.216015	15.0998052
hsa-miR-363*	33.397717	4.503005	11.88105335
