# Supplementary material for: miRNA Enriched in Human Neuroblast Nuclei Bind the MAZ Transcription Factor and Their Precursors Contain the MAZ Consensus Motif
Source: Front Mol Neurosci. 2017 Aug 21;10:259. doi: 10.3389/fnmol.2017.00259 (PMC5573442; doi:10.3389/fnmol.2017.00259)
Supplement: Supplementary file 2 [file Table_2.PDF]

Supplemetary table S2. Motif analysis of nucleus-depleted miRNA (<25% total expression)

miRNA name	Mature sequence	Contains motif?
**hsa-miR-342-5p**	AGGGGUGCUAUCUGUGAUUGA	**N**
**hsa-miR-25***	AGGCGGAGACUUGGGCAAUUG	**N**
**hsa-miR-129-3p**	AAGCCCUUACCCCAAAAAGCAU	**N**
**hsa-miR-602**	GACACGGGCGACAGCUGCGGCCC	**N**
**hsa-miR-572**	GUCCGCUCGGCG**GUGG**CCCA	**Y**
**hsa-miR-30b***	UGUAAACAUCCUACACUCAGCU	**N**
**hsa-miR-598**	UACGUCAUCGUUGUCAUCGUCA	**N**
**hsa-miR-148b**	UCAGUGCAUCACAGAACUUUGU	**N**
**hsa-miR-200c**	UAAUACUGCCGGGUAAUGAUGGA	**N**
**hsa-miR-218-2***	CAUGGUUCUGUCAAGCACCGCG	**N**
**hsa-miR-130b***	ACUCUUUCCCUGUUGCACUAC	**N**
**hsa-miR-135a***	UAUAGGGAUUGGAGCC**GUGG**CG	**Y**
**hsa-miR-92b***	AGGGACGGGACGCGGUGCAGUG	**N**
**hsa-miR-874**	CUGCCCUGGCCC**GAGG**GACCGA	**Y**
**hsa-miR-379**	UGGUAGACUAUGGAACGUAGG	**N**
**hsa-miR-941**	CACCCGGCUGUGUGCACAUGUGC	**N**
**hsa-miR-767-5p**	UGCACCAUGGUUGUCUGAGCAUG	**N**
**hsa-miR-346**	UGUCUGCCCGCAUGCCUGCCUCU	**N**
**hsa-miR-1225-5p**	GUGGGUACGGCCCA**GUGG**GGGG	**Y**
**hsa-miR-409-5p**	AGGUUACCCGAGCAACUUUGCAU	**N**
**hsa-miR-92a-1***	AGGUUGGGAUCGGUUGCAAUGCU	**N**
**hsa-miR-1303**	UUUAGAGACGGGGUCUUGCUCU	**N**
**hsa-miR-26b**	UUCAAGUAAUUCAGGAUAGGU	**N**
**hsa-miR-720**	UCUCGCUGGGGCCUCCA	**N**
**hsa-miR-532-3p**	CCUCCCACACCCAAGGCUUGCA	**N**
**hsa-miR-1301**	UUGCAGCUGCCUGGGAGUGACUUC	**N**
**hsa-miR-23b***	UGGGUUCCUGGCAUGCUGAUUU	**N**
**hsa-miR-1270**	CUGGAGAUAUGGAAGAGCUGUGU	**N**
**hsa-miR-1292**	UGGGAACGGGUUCCGGCAGACGCUG	**N**
**hsa-miR-193a-5p**	UGGGUCUUUGCGGGCGAGAUGA	**N**
