# Supplementary material for: miRNA Enriched in Human Neuroblast Nuclei Bind the MAZ Transcription Factor and Their Precursors Contain the MAZ Consensus Motif
Source: Front Mol Neurosci. 2017 Aug 21;10:259. doi: 10.3389/fnmol.2017.00259 (PMC5573442; doi:10.3389/fnmol.2017.00259)
Supplement: Supplementary file 3 [file Table_3.PDF]

Supplementary Table S3. Gene symbols of mRNA detected by Ago1/2 RIPseq.

Ago1 Unique	Ago2 Unique	Common
ABHD10	ACBD4	AAK1
ADAT1	ACTR1A	ABCC1
ADRA2A	ADAMTS19	ABCC5
ARHGAP35	ADAMTS5	ABHD2
ASXL2	ADCY9	ABL2
ATP8B2	ADPGK	ACACA
ATPAF1	AGAP2	ACLY
B4GALT1	AK4	ACSL4
B4GALT3	AKAP5	ACVR2A
BCL2L2	ANKH	ADAM12
BEGAIN	ANKRD40	ADAMTS4
BRWD3	ANKRD54	ADCY1
BTAF1	ANKS6	ADIPOR2
C12orf43	AP3M1	AEBP2
C14orf101	APBA1	AFAP1
C15orf41	APBB1	AFF3
C1orf21	APBB2	AFF4
C20orf27	ARC	AK3
C5orf22	ARF6	AKNA
C9orf3	ARFGEF2	AKT3
CAMK2N2	ARHGAP26	ALCAM
CCDC102A	ARHGAP28	AMOTL1
CDK16	ARL4C	ANKRD28
CDON	ARRDC3	ANKRD52
CERS5	ASB7	ANTXR1
CHRNA10	ATG13	AP1G1
CNNM1	ATP2B2	AP2B1
CNTNAP1	ATP5S	AP2M1
COPS7B	ATP6V0A1	APBA2
CREBBP	ATP6V0A2	APPBP2
CRTC3	ATXN1	ARFIP2
CTNS	ATXN7L3	ARG2
CTTNBP2NL	BAI1	ARHGAP39
CYB5R1	BBS4	ARHGEF12
CYTH2	BCAR3	ARID1A
DTNB	BCL2L11	ARID2
EGLN3	BCL7A	ARL3
ENG	BSN	ARMC8
ERBB4	BTG2	ARNT
FAM105A	C17orf85	ASB6
FAM120C	C1orf115	ASH1L
FAM160B2	C1orf198	ASPH
FAM193B	C20orf112	ASXL1
FAM3A	C21orf91	ATCAY

FANCA	C3orf70	ATF7
FBXL16	CABP7	ATG7
FBXL19	CADM3	ATG9A
FLNA	CAMK2D	ATP1B3
FMNL3	CAMK2G	ATP2B1
FOXJ2	CAMTA2	ATP6V1G1
FSTL4	CAPN12	ATRN
GABPA	CASKIN2	AZIN1
GDF11	CC2D1A	BACH2
GPCPD1	CCDC120	BAHD1
GPKOW	CCDC6	BAZ2A
GPSM1	CCDC86	BCAP31
GRIPAP1	CCDC92	BCL2
GTPBP1	CDC42EP4	BCLAF1
HK1	CDH11	BICD2
HLCS	CDK14	BMPR2
ICA1L	CDK18	BMS1
IGFBP5	CDK2AP2	BNIP2
INO80D	CDK5RAP2	BOC
ISCA1	CECR6	BRD2
KAT2A	CELF3	BRD7
KAT7	CELSR3	BRPF3
KCNK3	CHD1	BSDC1
KITLG	CHIC1	BTRC
KLHL12	CLCF1	C16orf52
L2HGDH	CLCN5	C16orf72
LDLRAD2	CLEC16A	C17orf96
LEMD2	CLTB	C1GALT1
LIN28A	CNIH2	C5orf63
LMO7	CNOT6L	C6orf106
LPPR2	CPD	C6orf89
LRFN4	CPEB2	CACHD1
LRP1	CRTAP	CACNA1B
LUZP1	CRY2	CACNA2D1
MAGED2	CSRP1	CACNA2D2
MANSC1	CWF19L1	CADM1
MAP3K9	CYB5RL	CADM2
MBD6	DAGLA	CALM1
MBNL2	DCAF15	CALM3
MICALL1	DCHS1	CALU
MKL1	DCLK1	CAMK1
MLLT3	DCTN5	CAMSAP1
MLXIP	DCUN1D3	CAPRIN1
MMS22L	DFFA	CARM1
MRAP2	DIP2B	CBL
MTMR3	DISP2	CBLL1

MTMR6	DNAL1	CBX5
MYH11	DOLPP1	CBX6
MYO19	DPF2	CCDC85C
NACC2	DRG2	CCDC88A
NCOA1	DTX3L	CCNJ
NFIX	DUSP3	CCNT2
NINJ1	DVL3	CCNY
NLGN2	DZIP1	CD276
NOL6	EBF1	CDK13
NR1D1	EDEM1	CDK6
NRN1	EFNB1	CELF5
NRXN1	EHD2	CENPO
NUP43	EIF1AD	CEP170
PAM	ELK1	CEP350
PDPK1	ENC1	CFL1
PFKFB2	ENDOD1	CGGBP1
PHF19	EPB41L4B	CHCHD3
PIGO	EPHB4	CHURC1
PITPNM3	EPHX1	CIC
PLCB3	EPS8L2	CISD1
PLD5	ETS1	CISD3
PLEKHG7	ETV5	CLCN3
PLEKHM3	ETV6	CLN6
POLR2F	FAM134A	CLVS1
PPP1R16B	FAM134C	CNBP
PRKACA	FAM161B	CNOT2
PRKRIR	FAM196A	CNTNAP2
PRMT6	FAM53C	COG5
PTBP1	FAM76A	COMMD10
PTDSS1	FAM84A	COPS2
PTPLA	FAM91A1	CPEB4
RAB3D	FAT3	CPLX1
RAD51D	FBXL20	CPLX2
RALBP1	FBXO24	CPSF2
RALGPS1	FBXO32	CPT2
RASA4	FBXO41	CRCP
RBM23	FBXO46	CREBZF
RNF121	FGD6	CRELD1
RNF185	FGFR2	CRIM1
SAR1B	FHL3	CSNK1E
SARM1	FNBP1	CSNK1G1
SCAI	FNDC3B	CSRNP3
SH3BGRL2	FOXN2	CSTF2T
SH3GLB2	FOXO4	CTDSPL
SH3KBP1	FSCN3	CUX1
SHARPIN	FUT9	CUX2

SLC16A2	G6PD	DCK
SLC1A4	GAL3ST3	DCP1A
SLC25A23	GALNT10	DCP2
SLC25A44	GALNT7	DCUN1D5
SLC2A4RG	GAS7	DCX
SMYD5	GATA4	DDAH1
SNAP91	GCH1	DDHD1
SOCS2	GCLC	DDT
SOX12	GIT1	DDX3X
SPATA2	GJB7	DDX42
SPRED1	GMEB1	DENND5A
SPTLC2	GNG12	DENND5B
STAG3L4	GNG2	DHDDS
STAT5B	GNS	DIEXF
STYX	GOT1	DIP2A
SYVN1	GPR124	DIP2C
TAPT1	GPRC5B	DMWD
TBC1D4	GRHL1	DNAJA1
TCF20	GRIA2	DNAJB1
TET3	GRIA3	DNMT3A
TLN1	GRIA4	DPP8
TMEM199	GSTM3	DPYSL2
TMEM214	GTDC1	DPYSL3
TMEM64	GTPBP3	DUSP16
TMEM91	HCFC2	DUSP8
TMOD2	HDX	DYRK1B
TNK2	HEG1	EAF1
TPP1	HIF3A	EEF1A1
TRIM46	HIP1R	EEF2K
TRIM66	HIST2H2BE	EFNB3
TSC1	HOXD9	EIF2B1
TSKU	HS6ST1	EIF4A2
TTC1	HUNK	EIF4G2
TULP4	ICK	EIF4H
U2AF2	IGDCC4	ELAVL1
UBE2W	IGF1R	ELAVL3
ULK2	IGSF10	ELN
UNC119B	IKZF4	ENAH
VAMP2	IP6K1	ENOSF1
VPS53	IQSEC1	EPB41L5
VSIG10L	ITGA3	EPM2AIP1
WBP1	ITGA4	EPT1
WBP2	ITGB3	ERC1
WDR52	ITGB8	ERRFI1
WDR5B	JHDM1D	ESRRG
XPO4	JUN	ETF1

ZC3H18	KALRN	EXOC5
ZFX	KCMF1	EXOC7
ZNF365	KCNA3	FAF2
ZNF507	KCNG1	FAM120A
ZNF740	KCNH4	FAM131A
ZSCAN22	KCNJ14	FAM168B
ZYX	KCTD15	FAM192A
	KCTD5	FAM3C
	KDM6A	FAM8A1
	KIAA0355	FBLIM1
	KIAA1522	FBXO11
	KIF1C	FBXO45
	KIFC2	FBXW2
	KLHL15	FGF2
	LDOC1L	FKBP3
	LEMD3	FNDC3A
	LENG8	FOXJ3
	LHFPL3	FOXN3
	LIMD1	FOXO3
	LMO4	FRMD4A
	LPCAT3	FSTL1
	LPIN2	FXR1
	LRP3	G3BP2
	LRRC14	GAB2
	LYST	GABRB3
	LZTFL1	GALNT2
	MAP1A	GATAD2B
	MAP1LC3A	GBF1
	MAP2K4	GDE1
	MAP6	GDPD1
	MAPK8IP3	GGA2
	MARCH4	GGA3
	MARCH9	GGCX
	MBD5	GIGYF1
	MCTP2	GJC1
	MEST	GLCCI1
	METTL17	GLG1
	MFI2	GLUL
	MINK1	GNAI2
	MMAA	GNB2
	MMP11	GORASP2
	MOCS1	GPATCH8
	MOSPD2	GPD2
	MPPED2	GPM6B
	MSI1	GPR137C
	MTHFD1	GPR161

MTUS2	GRB2
MXD1	GRK5
MYO1C	GSK3B
NACC1	H1F0
NCALD	H2AFX
NCOA5	HAND1
NDFIP2	HDAC8
NDRG1	HECTD1
NECAB3	HELZ
NEURL1B	HIF1AN
NEUROD1	HIPK1
NFASC	HIPK2
NFRKB	HIVEP1
NIPA2	HM13
NKD1	HMGA1
NLE1	HNRNPA1
NMNAT2	HNRNPA3
NOVA1	HNRNPF
NR3C1	HNRNPUL2
NT5M	HPCAL4
OAF	HSF2
OPA1	IL6ST
ORMDL2	IMPDH1
PAG1	INSIG2
PARD6B	INTS6
PATL1	IPO7
PBX1	ISL1
PBX2	ITCH
PCBP4	ITM2C
PCDH7	ITPR2
PDK3	KANK2
PEAR1	KCNAB1
PEX5L	KCNH1
PGAP3	KDM2A
PHC2	KDM5C
PHF15	KDM6B
PHF21B	KIAA0930
PHLDA3	KIF1A
PIM1	KIF1B
PKDCC	KIF3A
PLAGL2	KLF12
PLEKHA4	KLF6
POLDIP3	KLHDC3
POLI	KLHL24
POMT2	KLHL5
POU2F2	KMO

PPIL6	KPNA1
PPP1R9B	KPNA4
PPT2	KPNA6
PRELID2	KRIT1
PRKCI	LAMP1
PRR12	LAMTOR3
PRSS23	LCOR
PSKH1	LHFPL2
PTGFRN	LHFPL4
PTK2B	LIN7C
PTPN14	LMBRD1
PTPRO	LMO3
PTPRU	LPHN1
R3HDM2	LRCH1
RAB1B	LRP8
RABGGTA	LRRC59
RAP1GAP	LRRC8D
RAPGEF2	LSAMP
RARA	LUC7L3
RASAL2	M6PR
RASGRP2	MAP1B
RBM18	MAP3K13
RERE	MAP3K2
RGMB	MAP3K3
RHOBTB2	MAP4
RIC8B	MAP4K4
RIMKLA	MAP7D1
RND2	MAPK1IP1L
RNF114	MAPRE2
RNF220	38412
ROBO1	MARCKS
ROBO2	MARK2
RPS6KA2	MAT2A
RTN4R	MBTD1
RUNX1T1	MCC
SAP30L	MCL1
SCAF1	MCRS1
SCN8A	MED1
SCNM1	MED13L
SELT	MED17
SEMA4D	MED29
SEMA6A	MEGF9
SENP5	MEIS1
SESN2	MEIS2
SETD1A	METTL8
SETD8	MEX3A

SFMBT1	MIER1
SH2B3	MIER3
SH3GLB1	MKLN1
SH3PXD2B	MLL2
SHANK2	MLL3
SHB	MLL4
SHC3	MLLT4
SHISA5	MLLT6
SHROOM2	MRPL49
SIPA1L3	MTHFR
SKAP2	MTX3
SLC12A5	MXD3
SLC1A2	MXD4
SLC35E4	MYSM1
SLC8A2	NAA25
SLC9A6	NAMPT
SLCO3A1	NAPG
SLMO1	NARG2
SMARCD1	NDUFA4
SMG5	NEFL
SMURF1	NEK9
SNX19	NEO1
SNX21	NFAT5
SORCS3	NFATC3
SORL1	NFE2L1
SOX6	NFIB
SPATS2L	NFYA
SPRY4	NIPA1
SPTB	NIPBL
SRC	NIT1
SRF	NKRF
SRGAP3	NLGN4X
STAG1	NLK
STC2	NLN
STIM1	NONO
STK40	NOTCH3
STS	NPEPPS
STX1A	NR1H2
STXBP4	NR2C2
STXBP5L	NRARP
SYNGAP1	NRBF2
SYNGR3	NRCAM
SYNPO2L	NSD1
SYT13	NUBPL
TAOK3	NUDT16
TBC1D12	NUFIP2

TBC1D20	ODC1
TCEAL8	OLA1
TCTA	ONECUT2
THAP6	ORC2
THTPA	ORMDL3
TMC6	OSBPL7
TMEM127	OSBPL8
TMEM151A	OST4
TMEM201	OTUD5
TMEM222	PAIP2
TMEM231	PAPD5
TMEM63B	PAPOLG
TMEM63C	PCBP2
TMUB2	PCNX
TNFRSF12A	PDAP1
TNK1	PDE3A
TNKS2	PDGFRA
TP53RK	PDGFRB
TRAF3	PDHA1
TRIM39	PDSS2
TRIM59	PFKM
TRIM62	PGM3
TRIP10	PHC3
TSC22D3	PHF13
TSHZ2	PHF17
TSPAN33	PHF21A
TSPAN9	PHIP
TTLL5	PICALM
TXLNG	PITPNB
UBAP1	PKN2
UBE2L6	PMEPA1
UBE3B	POGK
UBE4A	POLDIP2
UPF1	POLR1D
USP31	POP4
USP46	POU2F1
USP50	PPM1A
UVRAG	PPP1CB
VAMP3	PPP1CC
VANGL1	PPP1R11
VASH2	PPP1R12A
VASP	PPP2R1A
VAV3	PPP2R3A
VCPIP1	PPP2R5C
VPS37C	PPP2R5D
VSTM2L	PRCD

WDFY2	PRDM2
WDR37	PRKAR2B
WDTC1	PRPF4
WIZ	PRRC2B
WWP2	PRRC2C
ZBTB34	PSIP1
ZC3H4	PTCH1
ZC3H7B	PTPN11
ZCCHC24	PTTG1IP
ZDHHC22	PUM1
ZDHHC7	PURB
ZFHX2	PVRL1
ZFP36L1	QKI
ZMYM3	QRICH1
ZNF26	QSER1
ZNF275	RAB11FIP4
ZNF282	RAB15
ZNF385A	RAB1A
ZNF502	RAB22A
ZNF609	RAD23B
ZNF689	RALGAPB
ZNF81	RAP1A
ZNHIT6	RASA1
	RASL11B
	RBFOX2
	RBM12
	RBM14
	RBM17
	RBM39
	RBM8A
	RBMS1
	RBMS2
	RBMS3
	RC3H1
	RCC1
	REEP3
	RGAG4
	RGS5
	RHOF
	RIC3
	RIC8A
	RICTOR
	RIMBP2
	RLIM
	RNF141
	RNF144A

RNF165
RNF169
RNF182
RNF20
RNF41
RNF44
RNF8
RORA
RPN2
RRP15
RTN3
RYK
SBK1
SBNO1
SDC1
SEC62
SEC63
SECISBP2L
SEPN1
40422
40787
38961
SERBP1
SERTAD2
SET
SETD6
SGK494
SH3PXD2A
SIPA1L2
SKIV2L2
SKP1
SLAIN2
SLC11A2
SLC12A2
SLC25A25
SLC26A2
SLC2A1
SLC2A12
SLC30A7
SLC30A9
SLC35C2
SLC38A2
SLC44A1
SLC50A1
SLC6A6
SLC7A14

SMAD2
SMAD4
SMAD5
SMAD9
SMARCA5
SMC1A
SMCR8
SMEK1
SMG1
SMPD3
SNTB2
SNX27
SOCS4
SOCS7
SPAG9
SPARC
SPEN
SPNS2
SRCIN1
SRGAP1
SRPK1
SRRM2
SSH1
SSH2
SSPN
SSR1
SSR2
SSR3
SSX2IP
ST8SIA2
STAT3
STIM2
STK16
STK35
STRN
SUPT16H
SURF4
SV2A
SYNC
SYNCRIP
SYNPO2
SYNRG
SYP
TAF9B
TANC2
TAOK1

TBL1XR1
TCEA1
TCEANC2
TCEB3
TCF12
TCF19
TEAD1
TEAD3
TFAP2B
TFDP2
THAP5
THSD7A
THY1
TLK1
TM9SF3
TMBIM6
TMEM115
TMEM120B
TMF1
TNPO1
TNRC6A
TNRC6B
TNRC6C
TOB2
TPM4
TRAK2
TRIT1
TSPYL5
TTC28
TTYH3
TUB
TUSC2
TWF1
U2SURP
UBE2G2
UBE2N
UBE2QL1
UBE2R2
UBE2Z
UBN2
UBR5
UBTD2
UBTF
UGGT1
ULK1
UNC5C

UNK
UPF3B
URM1
USF2
USP34
USP37
USP38
USP45
USP47
USP7
VANGL2
VAT1
VAV2
VCP
VMA21
VPS13D
WAC
WASF2
WDR1
WDR82
WHSC1
XRRA1
XYLT1
YAF2
YIPF5
YKT6
YOD1
YPEL5
YTHDF3
YWHAQ
ZBTB4
ZBTB44
ZCCHC3
ZFAND3
ZFAND5
ZFHX3
ZFHX4
ZFP106
ZFP14
ZFP36L2
ZFP90
ZFP91
ZFYVE27
ZHX3
ZKSCAN1
ZMAT3

ZMYM2
ZMYND11
ZNF24
ZNF260
ZNF280D
ZNF395
ZNF398
ZNF618
ZNF644
ZNF654
ZNF704
ZNF710
ZNF711
ZNRF1
ZSWIM6
